# Interaction of *Piriformospora indica* with *Azotobacter chroococcum*

**DOI:** 10.1038/srep13911

**Published:** 2015-09-09

**Authors:** Soubhagya Kumar Bhuyan, Prasun Bandyopadhyay, Pramod Kumar, Deepak Kumar Mishra, Ramraj Prasad, Abha Kumari, Kailash Chandra Upadhyaya, Ajit Varma, Pramod Kumar Yadava

**Affiliations:** 1 Applied Molecular Biology Laboratory, School of Life Sciences, Jawaharlal Nehru University, New Delhi-110067, India; 2 Amity Institute of Microbial Technology, Amity University, Noida-201303, Uttar Pradesh, India; 3 Amity Institute of Biotechnology, Amity University, Noida-201301, Uttar Pradesh, India

## Abstract

Microbial communities in rhizosphere interact with each other and form a basis of a cumulative impact on plant growth. Rhizospheric microorganisms like *Piriformospora indica* and *Azotobacter chroococcum* are well known for their beneficial interaction with plants. These features make *P. indica /A. chroococcum* co-inoculation of crops most promising with respect to sustainable agriculture and to understanding the transitions in the evolution of rhizospheric microbiome. Here, we investigated interactions of *P. indica* with *A. chroococcum* in culture. Out of five *Azotobacter* strains tested, WR5 exhibited growth-promoting while strain M4 exerted growth-inhibitory effect on the fungus in axenic culture. Electron microscopy of co-culture indicated an intimate association of the bacterium with the fungus. 2-D gel electrophoresis followed by mass spectrometry of *P. indica* cellular proteins grown with or without WR5 and M4 showed differential expression of many metabolic proteins like enolase-I, ureaseD, the GTP binding protein YPT1 and the transmembrane protein RTM1. Fungal growth as influenced by bacterial crude metabolites was also monitored. Taken together, the results conform to a model where WR5 and M4 influence the overall growth and physiology of *P. indica* which may have a bearing on its symbiotic relationship with plants.

In natural terrestrial ecosystems, almost all plants intimately associate with rhizospheric microorganisms^1^,^2^. These microorganisms such as mycorrhizal fungi, symbiotic nitrogen-fixing bacteria and some other free living plant growth promoting rhizobacteria (PGPR) are well known to contribute to soil fertility and crop production^3^,^4^. Mycorrhizal fungi and nitrogen fixing rhizospheric bacterial associations with plants are some of the best explored examples of mutualistic symbiosis. These relationships benefit the plants agronomically by increased growth, yield and ecologically improved fitness (tolerance to biotic and abiotic stresses). Rhizospheric organisms can grow, propagate and interact not only as individual cells, but also as multi-tropic communities. Previous reports on interaction of plants with mycorrhizal fungi and associated bacteria revealed that bacteria can be associated with fungal spores^5^ and hyphae^6^,^7^,^8^. The interactions of various rhizospheric microorganisms either separately or their combined impact on plants have been studied. Use of *L. bicolor* with mycorrhiza helper bacterial strains of *Pseudomonas* in combination has been shown to improve plant growth^9^. Sometimes, the bacteria and mycorrhizal fungus interact synergistically to mobilize soil PO_4_^3−^to the plant root through solubilization or mineralization^10^,^11^,^12^. Occasionally, specific rhizobacteria are also known to affect the pre-symbiotic stages of AM development^10^. This highly specific host–microbe interaction might be due to active molecular communication and physiological association among microbial multi-tropic communities^13^. However, different interactions among rhizospheric microorganisms are also crucial for the development of sustainable strategies for soil fertility and crop production. *Piriformospora indica* is an AM like fungus, originally isolated from the Thar Desert in India^14^. It interacts beneficially with a broad spectrum of plants and is known to enhance plant growth, biomass production, drought tolerance, salt tolerance, phosphorus acquisition and resistance to pathogens^15^,^16^,^17^,^18^,^19^,^20^. In interaction with *Oncidium* orchid roots, it alters expression of certain miRNA for growth promotion and developmental reprogramming and enhances stress resistance^20^.

The interaction of mycorrhizal fungi with plants is affected in various ways by other microorganisms, especially by rhizospheric bacteria. Mechanism of the symbiosis of bacteria with the fungus can be investigated by using techniques taking global view of proteomic, transcriptomic and metabolomic profile of the microbes, which may reveal changes that are undetectable by other means. Transcriptomic analyses of ectomycorrhizal fungus *Laccaria bicolor* in interaction (beneficial, neutral and antagonistic) with soil bacteria led to identification of common classes of genes linked to cell-cell interaction, stress response and metabolic processes^21^. Sharma *et al.* (2008) demonstrated that association of *P. indica* with *Rhizobium radiobacter* promotes the growth of barley seedlings and developed systemic resistance to the powdery mildew fungus *Blumeria graminis*^13^. Nautiyal *et al.* (2010) established that tripartite interaction enhanced nodulation and growth of Chick pea^22^. Some rhizobacterial strains belonging to diverse groups (Gram-positive Actinobacteria and Gram-negative Proteobacteria) exert neutral, stimulatory and inhibitory effect on the *P.indica*^23^. Co-inoculation of a phosphate solubilizing bacterium *Pseudomonas striata* and *P. indica* showed a synergistic effect on chickpea^24^. These studies may reflect on the basis of different interactions of *P. indica* with rhizospheric bacteria for later use in agronomical application. *Azotobacter chroococcum* is a free living bacterium known to improve plant growth either through nitrogen fixation or other plant growth promoting traits^25^. Single inoculants of *A. chroococcum* were found to enhance the growth of bamboo shoots and maize plants by phosphate solubilization and phytoharmone production^26^. Dual inoculants, in the presence *Glomus fasciculatum* (AM fungus), significantly enhanced the growth of tomato plants when compared with plants colonized by *G. fasciculatum* alone^27^, while *A. chroococcum* enhanced root colonization and spore production by the mycorrhizal fungus. This mycorrhizal infection profoundly increased *A. chroococcum* population. Further Prajapati *et al.* (2008) reported that dual inoculation of rice plants with *A. chroococum* and *P. indica* has improved rice plant biomass production significantly when compared with plants with single inoculation i.e. *Azotobacter* or *P. indica*^28^. Although synergistic interaction between mycorrhizal fungi and *Azotobacter* has been known, the molecular basis of such interaction still remains obscure. We have therefore selected *P. indica* – mycorrhiza like fungus and *A. chroococcum* as a model system to study the interaction. This is important for better understanding of microbial communication followed by establishment of sustainable strategy for rhizosphere research in context with agriculture and natural ecosystems.

In this study, the effect of *A. chroococcum* on growth and physiology of *P. indica* and the impact of bacterial strains on cellular proteins of *P. indica* were investigated. In addition, the fungal growth was assessed in broth and plate assay by using both the individual bacterial cell free supernatants and ultra-filtrate containing crude metabolites. We demonstrate that growth of *P. indica* was modulated via signaling secondary metabolites of plant growth promoting *A. chroococcum* strains WR5 and M4 during interaction in co-culture. Such experiments can be used to evaluate the specific natural bioactive compounds produced during co-cultivation.

## Results

### Strain specific interaction between *Azotobacter chroococcum* and *Piriformospora indica*

**Figure 1:**
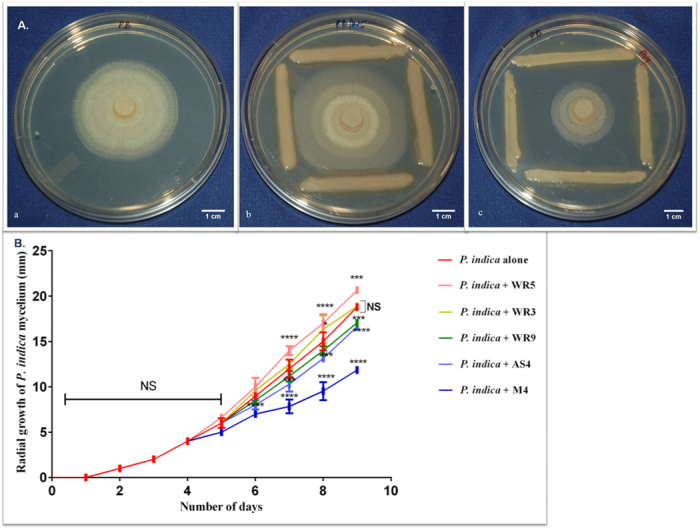
A. Visualization of the *A. chroococcum – P. indica* interaction in Hill and Kaefer agar plates in the presence and absence of *A. chroococcum* strains. (**a**) Control plate. (**b**) Interaction of *P*. *indica* with WR5. (**c**) Interaction of *P. indica* with M4. Figure 1B. Influence of *A. chroococcum* strains on the radial growth of *P. indica* inoculated in Hill and Kaefer medium. Statistical analysis was performed using GraphPad Prism software by two way analysis of variance (ANOVA), followed by Tukey’s multiple comparison test. Values represented as mean ± sd, n = 3. *P ≤ 0.01, ***P ≤ 0.001, ****P ≤ 0.0001 compared with *P. indica* alone. Error bar represents standard deviation (sd). NS, represents ‘not significant’.

Interaction of different strains of *A. chroococcum* with *P. indica* was studied in terms of their impact on growth of radially spreading fluffy mass of fungal hyphae, dry cell weight and sporulation (Fig. 1 and S1). After 72 hours of co-culture, the radius of hyphal disc of *P. indica* was 7.0 ± 0.26 mm (P < 0.0001) in the presence of M4 whereas in isolation it was 8.96 ± 0.37 mm. At this stage no significant change in the growth pattern of *P. indica* was observed in interaction with strains WR5, AS4, WR3 and WR9 in comparison to the control (Fig. 1B). In another 24 hours of co-culture, *P. indica* responded differentially to all the strains of *A. chroococcum*. Highly significant response was observed in the presence of WR5 and M4. In response toWR5, the average radius of mycelial disc was 14.0 ± 0.5 mm (P < 0.0001) whereas in the presence of M4, it was 7.83 ± 0.76 mm (P < 0.0001). Similar trend was observed for the next 24 hours. After six days of interaction on plate, WR5 showed stimulatory effect on fungal growth while it was arrested in response to the strain M4 (Fig. 1A).

In terms of dry cell weight, co-culture with WR5 yielded 14.0 ± 0.19 g/L (P < 0.0001) whereas it was found to be significantly lower at 7.68 ± 0.88 g/L (P = 0.0126) in the presence of M4 as compared to the fungus grown in isolation (9.87 ± 0.28 g/L). Similar observation was made with respect to spore yield as well. In isolation, the fungus yielded 5.14 (±0.35) × 10^6^ spores/ml. Significantly higher sporulation with 7.37 (±0.33) × 10^6^ spores/ml (P < 0.0001) was observed in the presence of WR5. In presence of M4, spore yield was limited to 3.67 (±0.29) × 10^6^ spores/ml(P < 0.0001). WR3, WR9 and AS4 didn’t make significant difference in fungal dry cell mass and spore yield (Fig. S2). Based on these observations, WR5 strain was picked as a stimulatory strain and M4 was selected as an inhibitory strain in terms of growth of *P. indica* to study the underlying molecular mechanisms of their interaction.

### Electron microscopic observation of the interaction of *A. chroococcum* strains with *P. indica*

**Figure 2:**
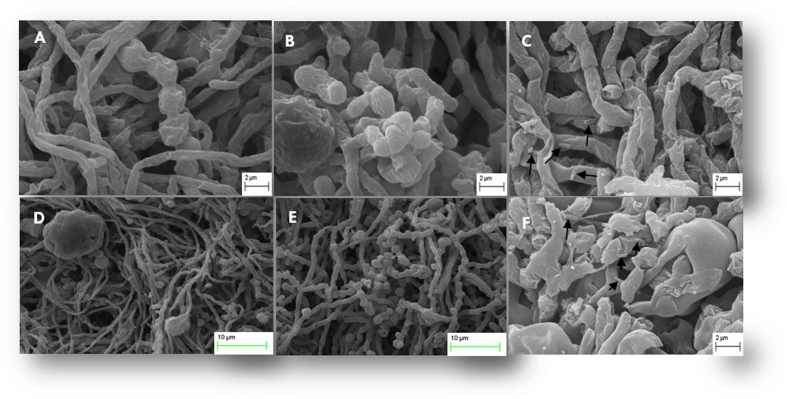
Scanning electron micrographs of *P. indica* morphology in isolation and co-culture with *A. Chroococcum* (WR5 and M4). (**A**) and (**D**): Control fungal mycelia appear to have normal hyphae, septa and conidia. Hyphae showed uniform tubular shape in all parts. (**B**) and (**E**): micrographs show a tendency of hyphal growth promotion induced by WR5. The main improvements are healthy fungal hyphae and more conidiation. (**C**) and (**F**): Hyphal growth affected by M4 showing damaged fungal hyphae with surface adhered rod-shaped bacteria (arrow in Fig. 3C) and lack of conidiation. Data shown is the representative of at least three independent sets of experiments.

After interaction individually with WR5 and M4 in broth, morphology of *P. indica* was viewed by Scanning Electron Microscopy (SEM). In the presence of the inhibitory strain M4 of *A. chroococcum*, the hyphae were found to be highly deformed, inflated, disaggregated and formed a loose mass. The hyphal network does seem to be uneven and clumpy. This could be attributed to the physical adherence of rod-shaped bacteria on the surface of mycelia as can be seen in Fig. 2C,F. In the presence of the stimulatory strain WR5, the hyphal wall was found to be thick, intact and hyaline. Intermittent globular structures either in cluster or in isolation, (1 to 5 globular structure/10 μm of hyphae), perhaps marking the initial stage of chlamydospore formation, are also seen (Fig. 2B,E).

**Figure 3:**
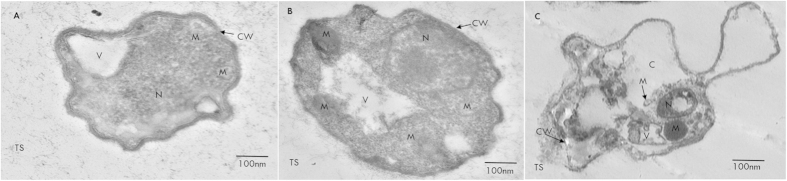
Transmission electron micrographs of *P. indica* in isolation and in co-culture with *A. chroococcum* (WR5 and M4) grown at 28 ± 1 °C. (**A**) Transverse section of control hypha showing cell wall (CW) mitochondria (M), vesicles (V) and nucleus (N). (**B**) Transverse section of hypha from co-culture with WR5 well organized hyphal cytoplasm, organelles and number of mitochondria. (**C**) Transverse section of hypha treated with M4 showing disorganization of hypha and cytoplasmic organelles and formation of membrane-bound vesicles. Data shown is the representative of at least three independent sets of experiments.

The Transmission Electron micrographs in Fig. 3A–C, show the effect of *A. chroococcum* on organization of hyphae. In the control hyphae, the cell wall was uniform and the plasma membrane was evenly spread. The cytoplasmic organelles such as nuclei, mitochondria, and vacuoles appeared well delineated. In co-culture with WR5 (Fig. 3B), morphological alterations were observed in the cell wall, organelles, diameter of hyphae and number of mitochondria, (3 to 5 mitochondria/100 nm). In co-culture with M4 the fungal mycelium showed cell-wall disorganization and depletion of hyphal cytoplasm. Other signs of degeneration included formation of vacuole-like membranous structure and small membranous vesicles. Cell organelles such as nuclei, endoplasmic reticulum and mitochondria were sparse (Fig. 3C).

### Effect of WR5 and M4 on fungal cellular proteome

**Figure 4:**
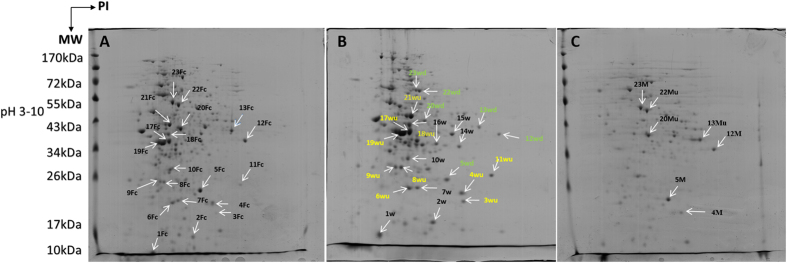
Representative 2DE profile of cellular proteins of *P. indica* in isolation or in co-culture with WR5 and M4 strains of *A. chroococcum*. Proteins from Control (**A**), WR5-treated (**B**) and M4-treated (**C**) cultures were separated by isoelectric focusing on linear IPG strips and run on 12% polyacrylamide SDS gel; numbers of differentially expressed spots correspond to protein identified by MALDI-TOF/MS and are listed in Tables 1 and 2.

In order to investigate the impact of WR5 and M4 on the cellular proteome of *P. indica* in co-culture, a profile was generated following two-dimensional gel electrophoresis for *P. indica* grown in absence (control) and presence of WR5 and M4. Gels from control and co-cultivated fungus were compared pair wise to detect differentially expressed spots (Fig. 4A–C). More than 500 protein spots were detected and matched in 2DE-gels between control and WR5-treated *P. indica*. Similarly, about 200 spots were detected and matched in between M4–treated and control fungus using Progenesis Same Spots image analysis software (version 4.5.4325.32621, Nonlinear Dynamics Ltd). Differentially expressed protein spots were reproducible and displayed within the pH range of 3–10 and mass range of 10–170 kDa. Spot images were analyzed pair wise between control and treatment to deduce fold expression level (Figs S3 and S4). A total of sixteen protein spots having significant changes in expression and present in sufficient amounts to be visible on the gel were selected for identification using MALDI-TOF/MS (Fig. S5). The proteins identified are listed in Tables 1 and 2. Of the sixteen protein spots chosen, ten were up-regulated, and six were down-regulated upon interaction with WR5 (Table1, Fig. S3). Interestingly, in interaction with M4, seven of the chosen sixteen spots seemed to have differential expression. Of these, four proteins were down-regulated and three proteins were up-regulated (Table 2, Fig. S4). Spots 3Wu, 4Wu/4M, 8Wu, 9Wu, 11Wu, 12Wd/12M, 13Wd/13Mu and 23Wd/23M were identified as uncharacterized conserved proteins of unknown function. Spots 6Wu, 17Wu/17Fc, 18Wu/18Fc, 19Wu/19Fc and 21Wu/21Fc (Fig. 4A–C and Tables 1 and 2) represent proteins involved in metabolic pathways. Spot 6Wu was identified as YPT-1 protein whereas Spot 17Wu and 17Fc was identified as UreD. The spots 18Wu and18Fc were identified as U3 small nucleolar RNA-associated proteins. They probably function as small nucleolar ribonucleoproteins in pre-ribosomal RNA processing reactions or in other biological function. This protein represents one of the few described so far for *Coprinopsis cinerea*. Spot 19Wu was found to be related to RTM1 protein. Spot 21Wu was Enolase I. Spots 5Wd/5M were identified as a G-protein alpha subunit of *Laccaria bicolor*, probably involved in signaling for cell growth. Spots 20Wi/20Mi resemble a protein involved in mitotic chromosome segregation and 22Wd/22Mu were identified as proteins mainly involved in signaling pathways that regulate cell division.

**Figure 5:**
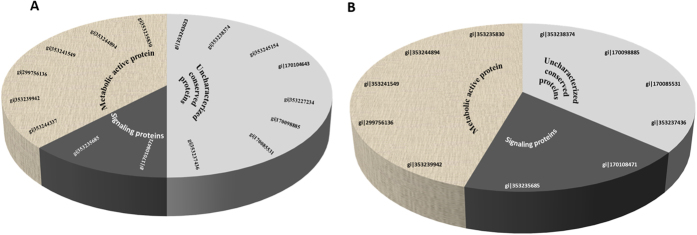
**A** schematic presentation of the distribution of functional categories of the identified cellular proteins of *P. indica* co-cultured with WR5 and M4. (**A**) A total of sixteen differentially expressed proteins were identified in WR5-treated condition. (**B**) A total of eleven differentially expressed proteins were identified in M4-treated condition. The percentage and numbers of proteins of the individual functional categories are shown.

All proteins were categorized into classes based on their biological function when searched in NCBI database through Mascott search engine (Fig. 5A,B). In interaction with WR5, the most highly represented class of eight proteins (50%) consists of uncharacterized conserved proteins of unknown function. Six proteins (37.5%) are involved in metabolic pathways for growth regulation. The final class consisting of two of the sixteen proteins (12.5%) are involved in signaling pathways. Of the eleven proteins differentially expressed in co-culture of *P. indica* with M4, five proteins (45%) are metabolic proteins, four (36.36%) are uncharacterized conserved proteins and remaining two (18.18%) are involved in signaling pathways. These results showed that WR5 up-regulated while M4, down-regulated the major metabolic cellular proteins of *P. indica*, (Tables 1 and 2).

### Effect of secondary metabolites of WR5 and M4 on growth of *P. indica*

**Figure 6:**
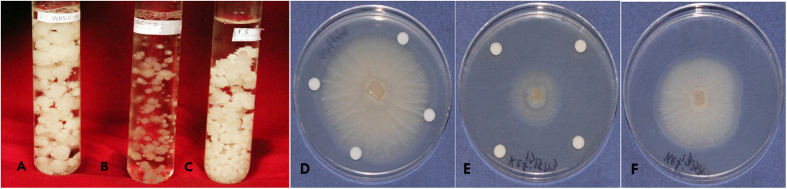
A Comparison of effects of culture filtrates of WR5 and M4 strains on the growth and development of *P. indica* with (A) WR5 culture filtrate, (B) M4 culture filtrate, (C) Control *P. indica,* in broth (D) Supernatant of WR5, (E) Supernatant of M4 and (F) Control *P. indica* (nine day old culture) on agar.

Effect of cell free bacterial supernatant of WR5 and M4 on growth of *P. indica* was studied. To evaluate the effect on growth of *P. indica*, 1% (v/v) crude cell free supernatant were added into 3-day old fungal culture broth separately. Among three experimental conditions, growth of fungal culture treated withWR5 cell free supernatant showed significant enhancement as compared to control culture (Fig. 6A,C). On the other hand, fungal growth was significantly decreased in the presence of M4 cell-free supernatant (Fig. 6B,C). Fungal growth was also assessed in terms of radial spread of fungal hyphae, using the 20 fold concentrates of ultra-filtrate microbial metabolites of each individual strain with control. Crude metabolites of WR5 stimulated while those from M4 inhibited growth when compared to control (Fig. 6D–F).

## Discussion

The physical associations established by mycorrhizal fungi with plant and bacterial cells can range from seemingly disordered polymicrobial communities to highly specific symbiotic associations. This tripartite association does exist in agricultural and forest ecosystems^2^,^29^. Many bacteria and fungi either in combination or in isolation have been shown to produce beneficial effects on plants. Interaction between *Pseudomonas putida* and *Glomus sp.* has been shown to promote plant growth by enhancing phosphate solubilization^30^,^31^. Certain signaling metabolites of *Streptomyces sp*. AcH505 have also been shown to stimulate the hyphal growth of *Amanita muscaria.* Similarly, volatile substances produced by some bark beetle-associated bacteria stimulate the growth of their symbiotic fungi^32^. Few PGPRs like *P. putida* IsoF promoted the growth of *P. indica* whereas *Pseudomonas fluorescens* WS5 and *Gluconacetobacter* sp. Comb19 inhibited fungal growth^23^. Though this kind of interactions is known to occur in the rhizospheric region, the exact nature of molecular interaction is yet to be elucidated.

To address this, we conducted interaction between *P. indica* and strains of *A. chroococcum* in axenic culture. Initial *in vitro* screening revealed different patterns of the growth modulating interactions of strains of *A. chroococcum*, with *P. indica*. Out of five *Azotobacter* strains, we identified two strains - WR5 and M4 which have the tendency to modulate the fungal growth. WR5 has maximal growth promoting effect and M4 strain has maximal growth inhibiting effect as seen in plate assay, dry cell weight content and spore yield. SEM and TEM results of *P. indica* did elucidate marked differences in the surface morphology and internal compartmentalization of cytoplasm and membranous organelles in interaction with WR5 and M4. Presence of healthy, thick hyphae in interaction indicates that WR5 supports fungal growth. Contrasting observations have been made in the presence of M4, where the hyphal architecture has been highly deformed. The cytoplasm has been disorganized in comparison to control. This suggests that in presence of M4, the fungus is metabolically less active. The interaction in agar and in broth is probably different in terms of the media environment, and the separation of the organisms in agar cultures, instead of direct association in broth. To resolve these interactions experiments with co-cultures in sterilized soil based substrates or perhaps in a clay variant such as vermiculite or perlite would more closely mimic the natural conditions.

**Figure 7:**
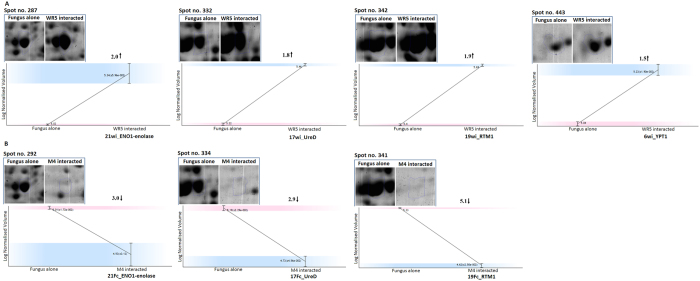
An analysis of the class “metabolic -proteins” in *P. indica* co-cultivated withWR5 (A) and M4 (B).

**Figure 8:**
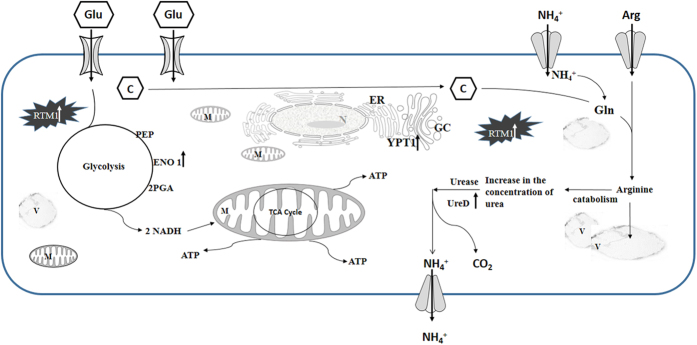
A hypothetical model showing changes in the carbon and nitrogen flux of *P. indica* in response to its interaction with WR5. Presence of WR5 enhances carbon pool in the mycelium triggering inorganic/organic nitrogen influx. This nitrogen is assimilated via glutamine synthetase/glutamate synthase cycle into arginine. Arginine is fed in the urea cycle; urea is further broken down to ammonia and carbon dioxide which is released. Stimulation in the expression of ENO1 and Ure D during carbon and in-/organic nitrogen influx has been indicated by up-arrows.

Further, to explore the mechanism of growth modulation of *P. indica* by both the strains, we demonstrated that WR5 and M4 lead to a specific modulation of protein expression in *P. indica*. In particular, *P. indica* co-cultured with WR5 showed an increase in the level of expression of some major metabolic proteins. The latter were down-regulated in the presence of M4. Based on a comparative analysis of major differentially expressed proteins of the class –‘Metabolically active-proteins’ (Fig. 7A,B), we present a hypothetical model in Fig. 8 suggesting the possible role of WR5 in stimulating the growth of *P. indica*. Up-regulation of both ENO1 and Ure D in the presence of WR5, suggests that WR5 could trigger efficient uptake of hexose sugar by the activation of several glucose transporters. ENO1 is one of the key regulatory enzymes of glycolytic pathway for generating reducing power for ATP synthesis. Ure D is one of the accessory proteins of the apoprotein UreABC which is a nickel-dependent regulatory enzyme involved in recycling of urea^33^,^34^. Ammonia generated by urease reaction is used as a source of nitrogen by the plant for its growth. This carbon supply generates the metabolic energy via glucose metabolism thus prompting the growth of *P. indica.* This has been well supported by increase in number of mitochondria as seen in co-cultures with WR5. As the model reflects, the energy generated and the carbon source may be further utilized in the active uptake processes of inorganic and organic nitrogen source by the mycelium further assimilating it into amino acid – Arginine^35^,^36^, which is loaded into the vacuoles and transported along the hypha. As a regulatory process, arginine is loaded into the anabolic arm of the urea cycle in order to be degraded, leading to an increased concentration of urea. In the presence of active urease, urea can be converted into ammonia and carbon dioxide^37^.

In conjunction with the activity of ENO1 and Ure D, YPT-1 and RTM1 proteins have unique importance in the growth of the fungus. YPT-1 does play a functional role in sporulation and the organization of the cytoskeleton during the vegetative state as it has been well documented in *S. cerevisiae*^38^. In addition, it has also been identified as global GTP-binding protein associated with trafficking of secretory vesicles between endoplasmic reticulum and Golgi complex^39^. *RTM1* is a membrane bound protein known to provide immunity and resistance to the fungus from the environmental toxic compounds^40^. Of the sixteen spots so identified, eleven correspond to *P. indica*, four to the closely related species *Laccaria bicolor* and one to *Coprinopsis cinerea*. The finding is in good agreement with the previous results of functional genomics analysis showing close phylogenetic relationship of *P. indica* with *Laccaria bicolor* and *C. cinerea*^41^. Proteomic analyses revealed that the metabolically active enzymes/proteins do play a key role in overall fungal physiology and growth modulation. Similar pattern of response was observed in interaction of *L. bicolor* with soil bacteria though different sets of genes/proteins were found to be involved in the interaction^21^.

The specific growth response of the fungus could be caused by specific bacterial metabolites released into the environment during co-cultivation. To test whether diffusible low molecular weight active signaling molecules elicit the fungal growth response, we performed fungus growth assessment in Petri dishes with twenty-fold concentrates of culture supernatants. The results obtained from these experiments suggest that the cell free culture supernatant might contain active metabolites for specific growth response of the fungus. The metabolites produced by WR5, contributed to the growth promotion of *P. indica* and a similar preparation from M4 showed inhibition of *P. indica* spore germination. *A. chroococcum* has two systems for nitrogen fixation: a conventional nitrogenase containing molybdenum (M nitrogenase) and an alternative nitrogenase involving vanadium (V nitrogenase)^42^. The bacterium has enhanced low-temperature nitrogen fixation activity to be associated with the Fe protein of V nitrogenase^43^. *A. chroococcum* produces phytohormones like auxins, gibberellins, cytokinins and ethylene which result in the promotion of plant growth and development^44^. These features make the *P indica*/*A. chroococcum* co-inoculation of non-legume crops most promising for optimal plant production. Along with *P. indica,* strains like WR-5 can be a better choice as nitrogen biofertilizer in cold region or the region like Thar Desert in India where plants experience a variety of stresses like drought, heat (summer) and cold (winter). A field trial of *A. chroococcum* strain M4 inoculation with maize demonstrated a significant increase in yields and saving of nitrogen fertilizer when applied in combination with farmyard manure^45^. The M4 strain can be further explored for its application as biocontrol for fungal pathogens associated with crops along with biofertilizer. The growth-promoting and inhibiting effects of *A. chroococcum* strains and their secondary metabolites on beneficial fungi have enormous potential for agronomical applications. Identification and validation of the potent molecular modulators (secondary metabolites) present in the culture supernatants, and studying its effect on cellular genes of the fungus by undertaking a genome-wide profiling of transcripts of *P. indica* will help establish the mechanism of such interactions.

Considerable effort has been made to understand the ecological significance of such bacterial fungal interactions in the rhizosphere. It has been found that even certain environmental changes could trigger free-living organisms to be mutualistic without necessitating adaptive co-evolution^46^. This is in good agreement with the experimental evidences for mutualistic association between free living organisms being induced by environmental conditions^47^. However, the degree of progression in the lines of mutualism seems to depend on species-specific traits^46^. To address this issue, further environmental studies supported by biochemical and molecular profile of such interactions can be undertaken.

The interaction of microorganisms with plant rhizosphere is not an autecological issue and has serious secondary impact on the performance of the plant vis-à-vis other organisms in its surroundings. Repeated selection for single traits in cultivars must have narrowed their interaction with microbiota in the rhizosphere. It has been argued that studies defining factors that determine the nature of microbiome would have to integrate results of systems-based approach in rhizospheric soil ecosystem. Plant species, soil type, agricultural practices, climatic factors, plant community structure and nature of biotic interaction of microbes with plants would together determine the course of plant-microbe co-evolution. The below-ground biological interactions influence biochemical constitution and behavior of above ground multitropic organisms including pests and pathogens. This illustrates the probable relevance of the present observations on differentially expressed proteins of *P. indica* and *Azotobacter* for developing cultivars for sustainable agriculture by rhizosphere engineering^48^.

## Methods

### Bacterial, fungal strains and culture conditions

The fungus, *P. indica* was maintained on Hill and Kaefer agar medium at 28 ± 2 °C and was used in all the experiments. To study the interaction with *P. indica,* prescreened plant growth promoting strains of *A. chroococcum* were used. They are WR5, WR9, WR3, AS4 and M4. WR5, WR9 and WR3 have been isolated from the rhizosphere of *Triticum aestivum* (wheat). M4 has been isolated from the rhizosphere of *Zea mays* (maize) whereas AS4 was isolated from the rhizosphere of *Allium cepa* (onion). Strains were provided by Dr. A.K Saxena, Division of Microbiology, IARI, New Delhi. The strains were maintained in Jensen’s medium at 28 ± 2 °C prior to the interaction. The interaction was studied in Hill and Kaefer medium.

### Effect of *A. chroococcum* strains on mycelial growth of *P. indica*

The growth of *P. indica* in interaction with *A. chroococcum* was expressed in terms of radial expansion on agar plate, dry cell weight per litre of culture broth and spore yield per litre of culture medium. For the radial growth determination, Hill and Kaefer agar plates were initially inoculated in the centre with the fungal agar plug (5 mm) obtained from a freshly grown culture plate of *P. indica*. It was later incubated at 28 ± 2 °C in the dark. On the third day of growth, all the five strains (WR5, WR9, WR3, AS4 and M4) were streaked in the periphery of each plate separately. Control was maintained, where *P. indica* was grown in isolation. The plates were further incubated for six days and daily observations were made for the radial growth of *P. indica* growing with or without *A. chroococcum*.

For determination of dry cell weight and spore yield, 250 ml Erlenmeyer flasks with 100 ml of Hill and Kaefer medium were inoculated with *P. indica* spores (5000spores/ml). The flasks were incubated at 28 ± 2 °C in the dark at 130 rpm. For interaction, secondary culture of *A. chroococcum* grown till OD 0.4(600 nm) were added individually into the six day old culture of *P. indica.* They were co-cultured for additional three days. Separate controls were maintained where *P. indica* was grown in isolation. On the ninth day, fungal biomass was filtered and washed three times with 1X PBS and pelleted by centrifugation at 7000 rpm for 10 min. Further, dry cell weight and spore yield were determined according to Kumar *et al.*, (2011)^49^.

### Scanning and Transmission Electron Microscopy

For Scanning electron microscope observations on the effect of bacteria on growth and morphology of *P. indica*, the fungus was grown in Hill-Kaefer medium at 28 ± 2 °C for six days. For interaction, secondary culture of *A. chroococcum* strains (WR5 and M4) with A_600_ of 0.4, were individually added into six day old fungal cultures (three replicates each). The co-cultures were incubated for additional 3 days with control fungus. On day nine the control and co-cultured mycelium were washed three times with 1X PBS and fixed with 2.5% glutaraldehyde (Sigma) in PBS (pH7.4) and post -fixed in 1% osmium tetroxide (Sigma); they were later washed by 0.1 M sodium cacodylate buffer, pH 7.4 (Sigma). After eliminating the remaining osmium tetraoxide, the samples were dehydrated in graded series of ethanol. The specimens were then dried in the critical point dryer (Emitech, k850). The dried specimens were mounted onto stubs by double-sided carbon tape. The specimens were coated with a thin layer of gold by a Polaron SC 502 sputter coater, and were examined in the Scanning Electron Microscope (Carl Zeiss EVO40). For the transmission electron microscope observation, after the dehydration step, the fixed mycelium was embedded in Epoxy resin cy212 and the small blocks of mycelium were sectioned with an ultra-microtome (Leica EMUC-6). The ultra-thin sections were then analyzed using TEM (Jeol 2100 F).

### Protein extraction

*P. indica* spores were germinated and grown in 100 ml of Hill-Kaefer’s broth at 28 ± 2 °C for six days. For interaction, secondary culture of *A. chroococcum* strains WR5 and M4 (OD_600_ of 0.4), were individually added into six day old fungal cultures and again co-cultured for additional three days with control fungus. On the ninth day, control and co-cultivated mycelium was washed three times with 1X PBS and pelleted at 7000 rpm for 10 min at 4 °C. For protein extraction, pellet (5.55 gm fresh weight) from each experimental condition with control fungus were individually ground to fine powder in liquid nitrogen using sterile pestle-mortar. The fine powder was collected into a cold 50 ml centrifuge tube and homogenized with 10% TCA in acetone with 0.07% 2-mecraptoethanol by vortexing for 10 min and incubated overnight at −20 °C. The precipitated protein fraction was centrifuged at 14,000 rpm for 20 min at 4 °C and pellet was collected. The pellet was washed twice with chilled 90% acetone containing 10 mM dithiothreitol (DTT) and twice with 80% chilled acetone containing 10 mM DTT and collected by centrifugation for 10 min at 14,000 rpm at 4 °C. Residual acetone was air-dried for 10 min at 4 °C and the precipitate re-suspended in protein solublization buffer [8M urea, 0.02% β-mecraptoethanol, 4% (w/v) CHAPS, 30 mM Tris-base, 1mM EDTA (pH8.0), 2% IPG –buffer (pH3-10), 0.1% SDS and 1mM PMSF] using sonication with a sonic-probe. Once the solution became homogenous, it was spun for 15 min at 14,000 rpm at 4 °C and the supernatant was collected and protein quantified by Bradford reagent using bovine serum albumin (BSA) as a standard.

### 2D-Electrophoresis

In order to perform 2D-gel electrophoreses 250 micrograms of total protein from each experimental group were suspended in rehydration buffer. The individual protein mixtures were pipetted in a 13 cm ceramic strip holder in which an immobilized linear pH gradient (IPG) strip, pH 3–10, 13 cm (GE Health care) was placed gel side down ward. The strips were rehydrated overnight at room temperature and focused by increasing voltage over 8 h from 100 to 10.000 V. For the second dimension the strips were equilibrated in equilibration buffer and placed on a 12% polyacrylamide SDS PAGE gel. Electrophoresis was performed at room temperature using the Bio-Rad-power-PAC 200 at 100 V. Protein spots were visualized by, staining with Coomassie Brilliant Blue G250 in methanol (40%) acetic acid (10%) and destained with distaining solution (30% methanol, 10% acetic acid).

### Analysis of 2D-PAGE

Three independent 2DE gels were scanned using an UMAX power Look 2100xL Image scanner (GE Health care). To identify spots of interest, gels from pure fungal culture (control) were compared pair-wise with the co-cultured fungus (*P. indica*-with WR5 and *P. indica*-with M4). The Progenesis Same Spots image analysis software version 4.5.4325.32621, Nonlinear Dynamics Ltd, UK, allowed us to perform both a qualitative (presence vs absence) and a quantitative analysis. For identification of differentially expressed proteins, relative spot volumes of the three replicate gels were compared and the quantity of each spot was normalized with respect to the total spot volume of the 2-DE gel. The difference in spot volume was used as a measure of change in protein expression and significantly different intensities (P < 0.05) of at least 1.2 fold difference between two experimental groups were considered significant for further analysis.

### Sample preparation and protein identification

Protein spots were excised, washed with proteomic grade de-ionized water and identified by mass spectrometry using MALDI-TOF mass spectrometer (Bruker Daltonic). The gel piece containing the protein were destained in 100% acetonitrile in 100 mmmM NH_4_HCO_3_ for 15 min at 37 °C and shrunken in 100% ACN for 5 min at room temperature. After destaining, the gel particles were soaked in 10 mM DTT in 100 mM NH_4_HCO_3_ for 45 min at 56 °C and alkylated with iodoacetamide (55 mM in 100 mM NH_4_HCO_3_ buffer). After removal of residual solvent gel-particles were subject to in gel-trypsin digestion (10 ng in 25 mM NH_4_HCO_3_ buffer) at 37 °C overnight. Subsequently tryptic peptides were extracted with 1% TFA plus 100% ACN (1:1) and concentrated under vacuum (Speed Vac) at 37 °C. Peptides were eluted with 0.7 μl MALDI matrix solution (4 mg α-cyano 4-hydroxy cinnamic acid (CHCA) in 60% ACN containing 0.1% TFA) and directly deposited on the MALDI target plates (Applied Biosystems). All mass spectra were recorded in positive reflector mode. First MS spectra were acquired from the standard peptides on each calibration spot and the default calibration parameters of the instrument were updated. Subsequently, MS spectra were recorded for all sample spots on the MALDI target plate and internally calibrated using signals from autoproteolytic fragments of trypsin. Peptide mass MS/MS data were analyzed with MASCOT search engine against the NCBI nr database and with Fungus as the species searched, allowing one missed cleavage of trypsin per peptide, accuracy level of 150 ppm and mass tolerance of 0.3Da. Only matches with P < 0.05 for random occurrence were considered for identification.

### Preparation of secondary metabolites from bacterial culture broth for *P. indica* growth assay

To evaluate the bioactivity of the bacterial culture filtrate against fungal growth, we had initially cultivated both WR5 and M4 bacterial strains in Jensens medium separately. At an O.D. of 0.4, the culture was diluted to 10^−3^X in the Hill-Kaefer broth and cultured for three days at 28 ± 2 °C. Cells were harvested by centrifugation (13,000 rpm for 20 min at 4 °C), and supernatants were filter sterilized through 0.22 μm filter (Millex, 33 mm, Millipore). After filter sterilization, the culture filtrates were used for mycelium growth assay in three day-old broth culture containing mycelium in the culture flask by adding 1% culture-filtrates per day for eight days. For plate assay, filter sterilized bacterial supernatants were again passed through the 1kDa cut-off Microsep advance centrifugal device (Pall Corporation) for isolation of active bacterial metabolites. The ultra-filtrates were concentrated up to 20-fold by vacuum evaporation at 37 °C. The concentrated metabolites were used for fungal growth assay in petri dishes by adding 20 μl volume into each disk in the three day-old culture on Hill and Kaefer agar plates, which was continued till eight days.

### Statistical analysis

To study the effect of *A. chroococcum* strains on the radial growth of *P. indica*, statistical comparison was made using two way analysis of variance (ANOVA) followed by Tukey’s multiple comparison tests. Whereas one way analysis of variance (ANOVA) was conducted followed by Dunnett’s multiple comparison test for dry cell weight and spore yield determination of *P. indica* in the presence and absence of *A. chroococcum* strains. GraphPad Prism v6.04 was used for the analysis. The data is reported as mean ± sd.

## Additional Information

**How to cite this article**: Kumar Bhuyan, S. *et al.* Interaction of *Piriformospora indica* with *Azotobacter chroococcum*. *Sci. Rep.* **5**, 13911; doi: 10.1038/srep13911 (2015).

## Supplementary Material

Supplementary Information

## Figures and Tables

**Table 1:** List of differentially expressed proteins of *P. indica* co-cultured with *A. chroococcum* WR5, as determined by 2-DE and MALDI-TOF/MS.

**Spot No.**^a^	**Spot identifier number**^b^	**Protein name (Accession No)**^c^	**Organism**^d^	**Score**^e^	**MW/PI**^f^	**Sequence Coverage (%)**^g^	**No of peptides matched**^h^	**Role in biological process**	**P value**^i^	**Fold change**^j^
3Wu	457	Hypothetical protein PIIN_09126_ gi|353243623	*P. indica*	25	11.647/ 10.58	30.4	2	NA	5.008e-007	4.3 ↑
4Wu	444	hypothetical protein PIIN_04261_ gi|353238374	*P. indica*	20	22.836/9.10	15.2	2	NA	2.182e-006	1.8 ↑
6Wu	443	Related to YPT-1 GTP-binding protein ypt1 _gi|353244337	*P. indica*	55	24.968/5.47	11.5	3	Sporulation, organization of the cytoskeleton during vegetative growth in *S. cerevisiae* and associated with trafficking of secretory vesicles between ER to golgi complex.	1.017e-005	1.5 ↑
8Wu	440	hypothetical protein PIIN_10216_ gi|353245154	*P. indica*	56	25.862/9.8	8.0	3	NA	1.502e-005	1.7 ↑
9Wu	406	predicted protein _ gi|170104643	*Laccaria bicolor*	41	17.750/5.70	7.6	2	NA	7.334e-006	1.8 ↑
11Wu	416	Hypothetical protein PIIN_02973_ gi|353227234	*P. indica*	19	30.470/6.20	6.1	2	NA	6.459e-007	2.0 ↑
17Wu	332	Hypothetical protein PIIN_05768 _ gi|353239942	*P. indica*	31	36.860/9.65	8.0	4	A nickel dependent regulatory enzyme involved in recycling of urea.	5.093e-008	1.8 ↑
18Wu	320	u3 small nucleolar RNA-associated protein 11_ gi|299756136	*C. cinerea*	25	39.845/9.30	4.1	3	Probably function as small nucleolar ribonucleoproteins in pre-ribosomal RNA processing reactions or in other biological function.	2.579e-007	1.8 ↑
19Wu	342	related to RTM1 protein_ gi|353241549	*P. indica*	37	33.056/10.0	9.5	2	Provide immunity and resistance to the fungus againstenvironmental cytotoxic compounds.	2.899e-008	1.9 ↑
21Wu	287	Probable ENO1-enolase I (2-phosphoglycerate dehydratase) _gi|353244894	*P. indica*	98	43.573/5.3	23.7	6	Key regulatory enzyme that participates in glycolytic pathway for generating reducing powers for ATP synthesis.	1.079e-004	2.0 ↑
5Wd	428	G protein alpha-subunit _ gi|170108471	*Laccaria bicolor*	68	8.129/4.6	47.8	3	GTP binding protein,probably involved in signaling for cell growth.	3.752e-004	1.2 ↓
12Wd	330	predicted protein_ gi|170098885	*Laccaria bicolor*	53	33.900/6.1	17.5	3	NA	9.711e-006	1.6 ↓
13Wd	305	predicted protein _ gi|170085531	*Laccaria bicolor*	25	36.625/10.1	4.2	3	NA	0.005	5.3 ↓
20Wd	296	Hypothetical protein PIIN_01661_ gi|353235830	*P. indica*	58	39.858/10.0	20.0	3	Mitotic chromosome segregation	0.003	1.2 ↓
22Wd	207	Hypothetical protein PIIN_01521_ gi|353235685	*P. indica*	49	44.690/5.8	10.4	3	Involved in the process of signaling path way that regulate cell division.	2.952e-005	1.7 ↓
23Wd	198	Hypothetical protein PIIN_03309 _ gi|353237436	*P. indica*	45	57.965/6.80	11.4	3	NA	1.506e-004	1.6 ↓

^a^Spot numbers refer to the numbers on the 2DE gels shown in Fig. 5A,B.

^b^Protein spot identifier number.

^c^Protein accession number according to the MASCOT software.

^d,e,f,g,h^Name of the organism, score, theoretical MW/PI, sequence coverage, no of peptides matched according to the MASCOT software.

^i^P value according to progenesis samespots analysis.

^j^Protein fold change according to the 2D-PAGE analysis shown in Fig. S2. ‘Wu’ represents upregulated and Wd represents downregulated fungus treated with WR5. ‘↑’ and ‘↓’ Represents up and down regulation.

**Table 2:** List of differentially expressed proteins of *P. indica* co-cultured with *A. chroococcum* M4, as determined by 2-DE and MALDI-TOF/MS.

**Spot No**^a^	**Spot identifier number** b	**Protein name (Accession No)**^c^	**Organism**^d^	**Score**^e^	**MW/PI**^f^	**Sequence Coverage (%)**^g^	**No of peptides matched**^h^	**Role in biological process**	**P value**^i^	**Fold change**^j^
17Fc	334	Hypothetical protein PIIN_05768 _gi|353239942	*P. indica*	31	36.860/9.65	8.0	4	A nickel dependent regulatory enzyme involved in recycling of urea.	1.079e-005	2.9 ↓
18Fc	320	u3 small nucleolar RNA-associated protein 11_ gi|^29^9756136	*C. cinerea*	25	39.845/9.30	4.1	3	Probably function as small nucleolar ribonucleoproteins in pre-ribosomal RNA processing reactions or in other biological function.	3.589e-004	1.5 ↓
19Fc	341	related to RTM1 protein_gi|353241549	*P. indica*	37	33.056/10.0	9.5	2	Provide immunity and resistance to the fungus againstenvironmental cytotoxic compounds.	1.306e-007	5.1 ↓
21Fc	292	Probable ENO1-enolase I (2-phosphoglycerate dehydratase )_gi|353244894	*P. indica*	98	43.573/5.3	23.7	6	Key regulatory enzyme that participates in glycolytic pathway for generating reducing powers for ATP synthesis.	2.505e-004	3.0 ↓
4M	452	hypothetical protein PIIN_04261_ gi|353238374	*P. indica*	20	22.836/9.10	15.2	2	NA	0.141	1.9*
5M	422	G protein alpha-subunit _ gi|170108471	*Laccaria bicolor*	68	8.129/4.6	47.8	3	GTP binding protein,probably involved in signaling for cell growth.	0.235	1.5*
12M	331	predicted protein_ gi|170098885	*Laccaria bicolor*	53	33.900/6.1	17.5	3	NA	0.133	1.3*
13Mu	309	predicted protein _gi|170085531	*Laccaria bicolor*	25	36.625/10.1	4.2	3	NA	7.660e-006	1.5 ↑
20Mu	301	Hypothetical protein PIIN_01661_ gi|353235830	*P. indica*	58	39.858/10.0	20.0	3	Mitotic chromosome segregation.	0.005	1.3 ↑
22Mu	210	Hypothetical protein PIIN_01521_ gi|353235685	*P. indica*	49	44.690/5.8	10.4	3	Involved in the process of signaling path way that regulate cell division.	0.001	1.5 ↑
23M	198	Hypothetical protein PIIN_03309 _gi|353237436	*P. indica*	45	57.965/6.80	11.4	3	NA	0.466	1.5*

^a^Spot numbers refer to the numbers on the 2DE gels shown in Fig. 5A,C.

^b^Protein spot identifier number according to the progenesis.

^c^Protein accession number according to the MASCOT software.

^d,e,f,g,h^Name of the organism, score, theoretical MW/PI, sequence coverage, no of peptides matched according to the MASCOT software.

^e^P value according to progenesis samespots analysis.

^f^Protein fold change according to the 2D-PAGE analysis of progenesis samespots analysis software shown in Fig. S3. ‘Fc’ represents Fungus control, ‘Mu’ represents upregulated and Md represents downregulated fungus treated with M4. ‘↑’ and ‘↓’ Represents up and down regulation. *Represents no significant changes in the fold level.
