# Supplementary material for: Interaction of Piriformospora indica with Azotobacter chroococcum
Source: Sci Rep. 2015 Sep 9;5:13911. doi: 10.1038/srep13911 (PMC4563567; doi:10.1038/srep13911)
Supplement: Supplementary Information [file srep13911-s1.pdf]

Interaction of *Piriformospora indica* with *Azotobacter chroococcum*

Soubhagya Kumar Bhuyan[1], Prasun Bandyopadhyay[2], Pramod Kumar[1], Deepak Kumar Mishra[1], Ramraj Prasad[1], Abha Kumari[1], Kailash Chandra Upadhyaya[3], Ajit Varma[2] & Pramod Kumar Yadava[1*]

Supplimentary data

Figure S1. Visualization of the *A. chroococcum – P. indica* interaction in Hill and Kaefer agar plates in the presence and absence of *A. chroococcum* strains. (A) control plate. (B) interaction of *P. indica* with WR3. (C) interaction of *P. indica* with WR9. (D) interaction of *P. indica* with AS4.

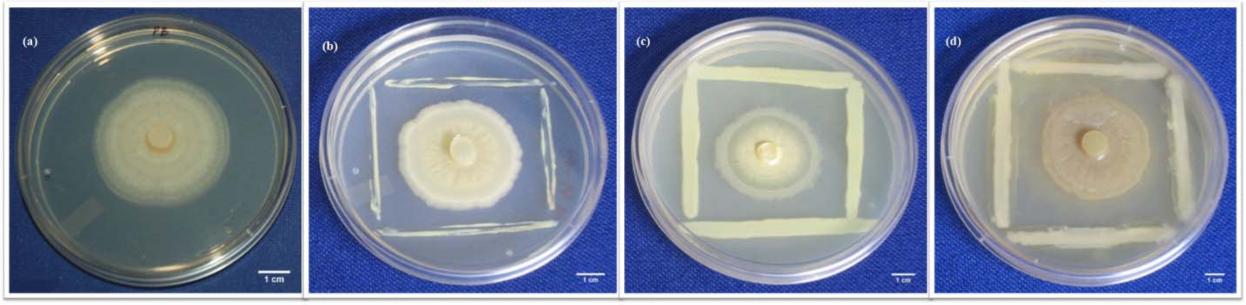

Figure S 2. Influence of *A. chroococcum* strains on the (A) dry cell weight and (B) spore yield of *P. indica* in Hill and Kaefer liquid medium. Statistical analysis was performed using GraphPad Prism software by one way analysis of variance (ANOVA), followed by Dunnett's multiple comparison test. Values represented as mean ±sd, n=3. **P≤0.01, ****P≤0.01 compared with *P. indica* alone. Error bar represents standard deviation (SD).

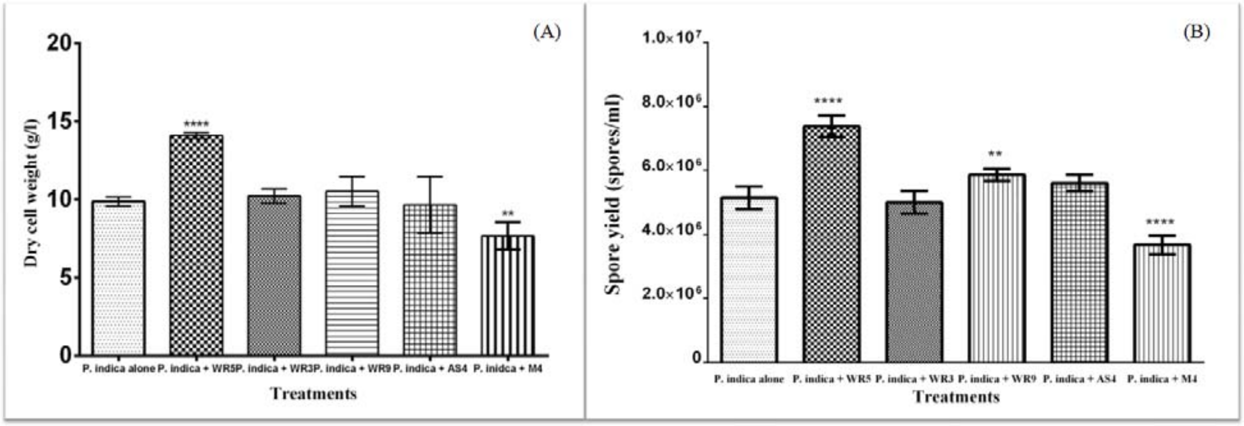

Figure S3. A list of protein spots identifier number, that exhibit change in expression fold level was analyzed pairwise between control and WR5-treated *P. indica* using Progenesis SameSpots analysis software.

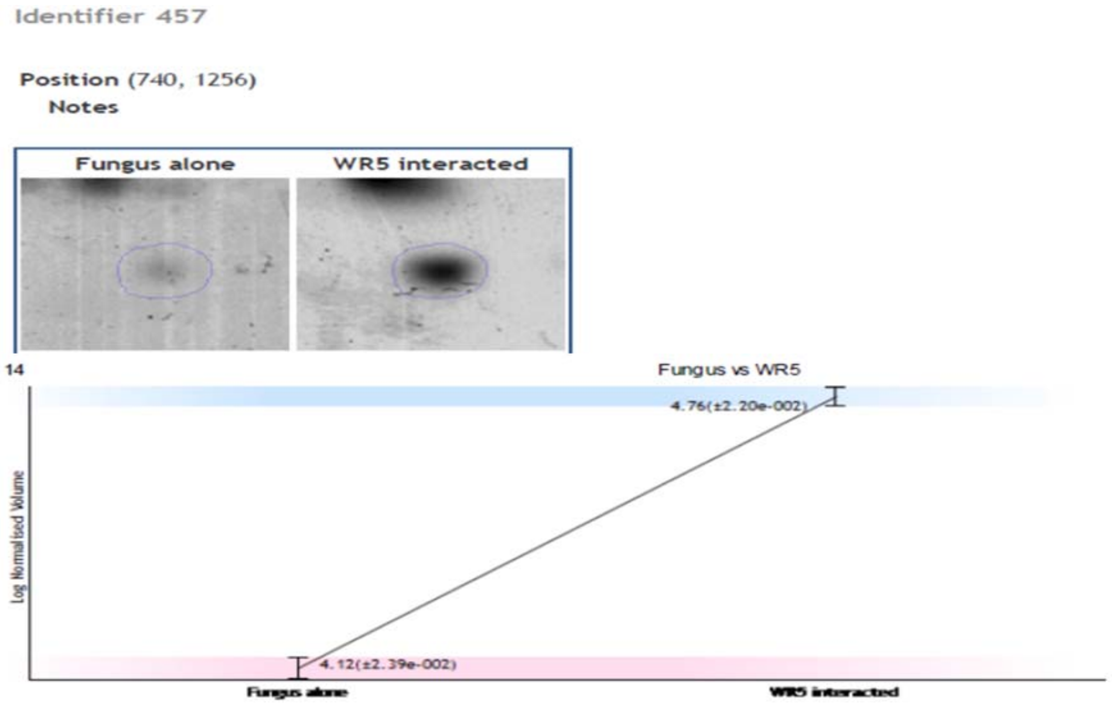

Identifier 444

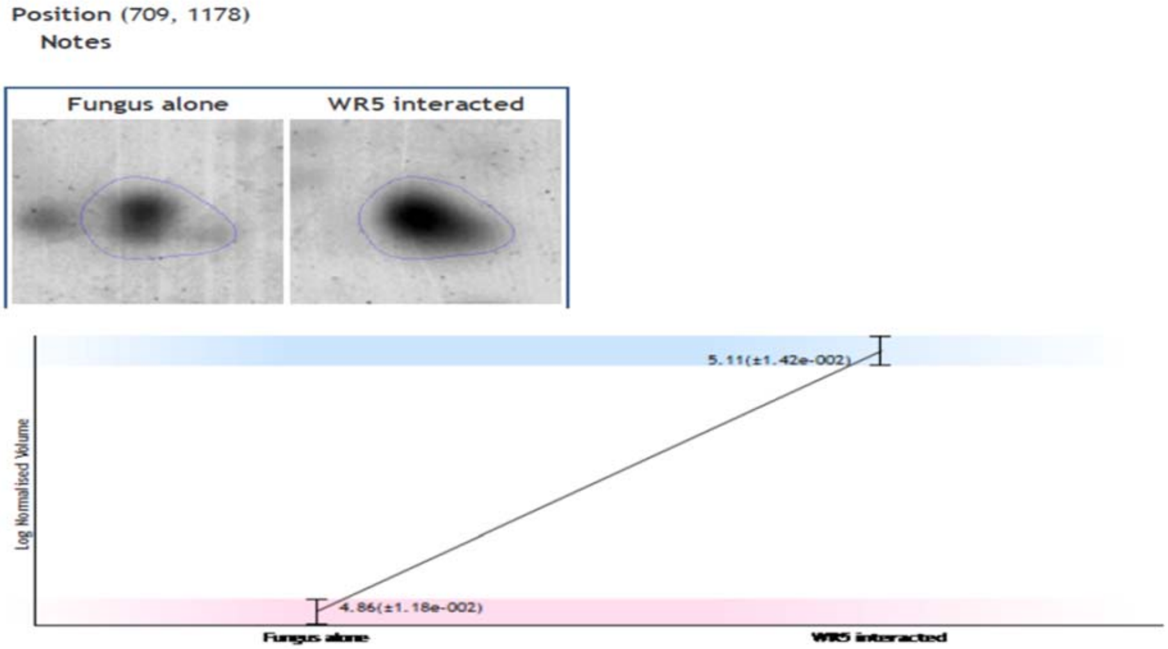

Identifier 443

Position (395, 1177)

Notes

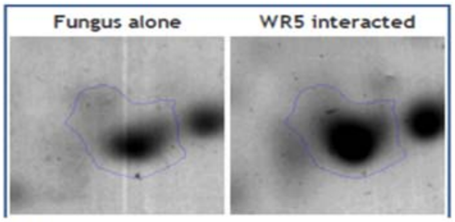

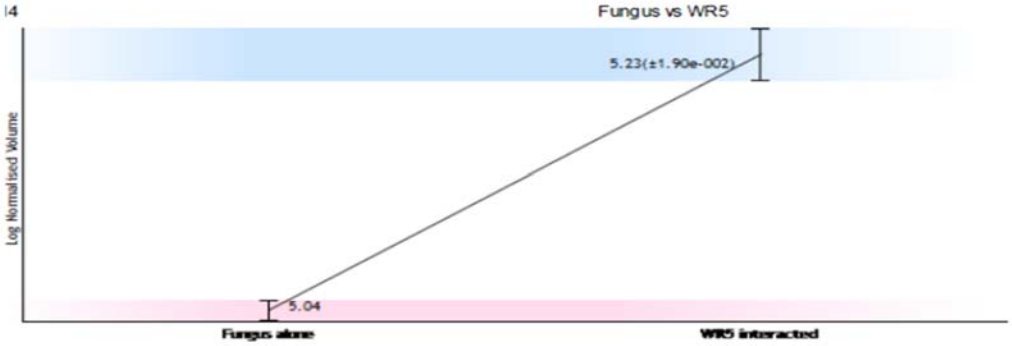

Identifier 440

Position (439, 1160)

Notes

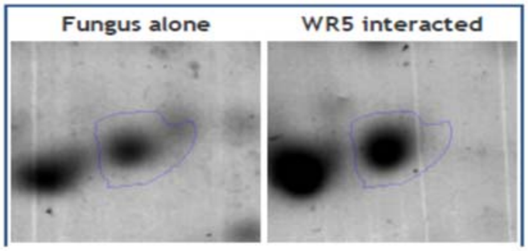

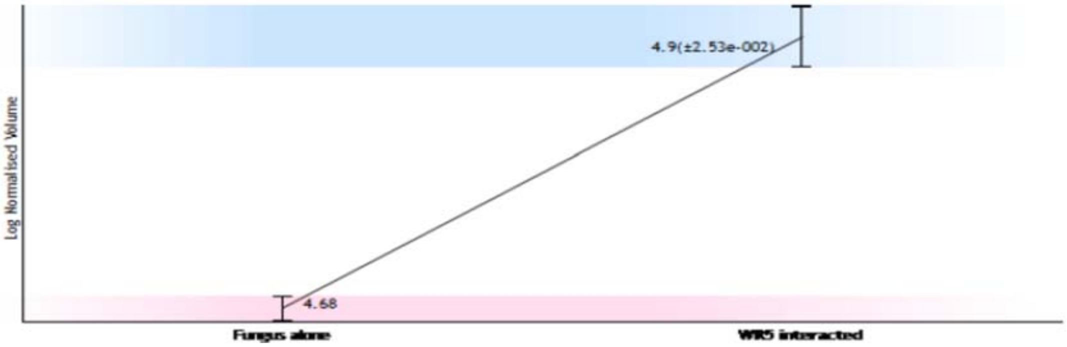

Identifier 406

Position (287, 999)
Notes

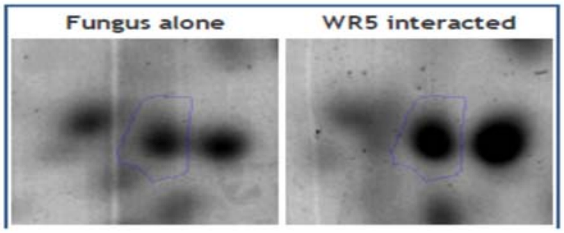

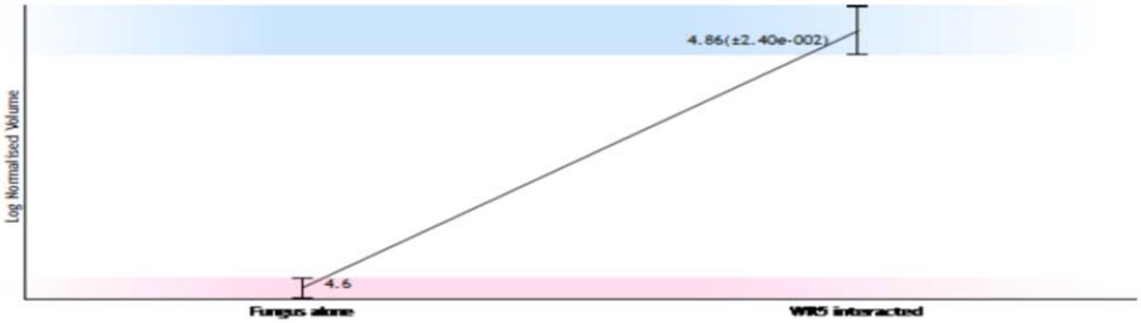

Identifier 416

Position (927, 1037)
Notes

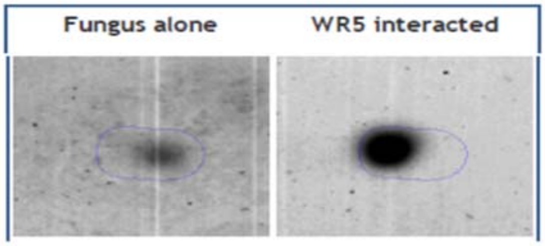

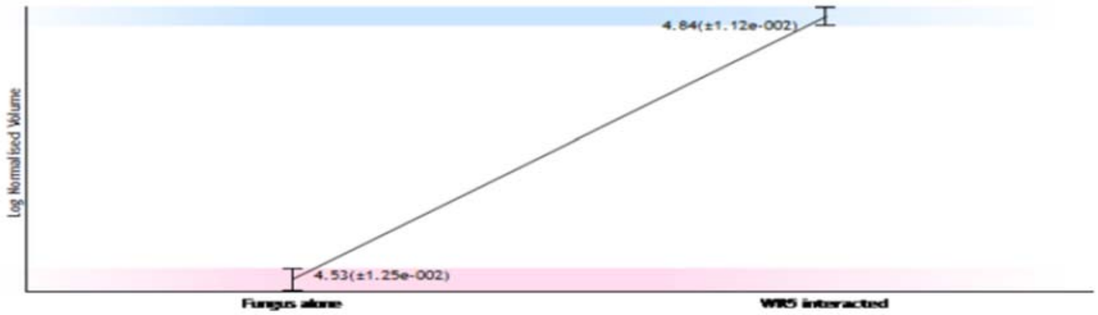

Identifier 332

Position (368, 710)
Notes

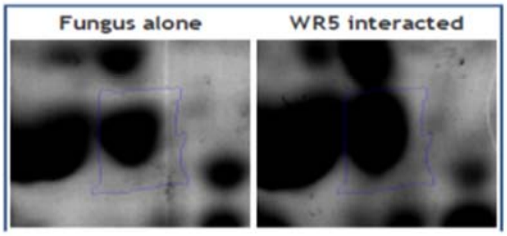

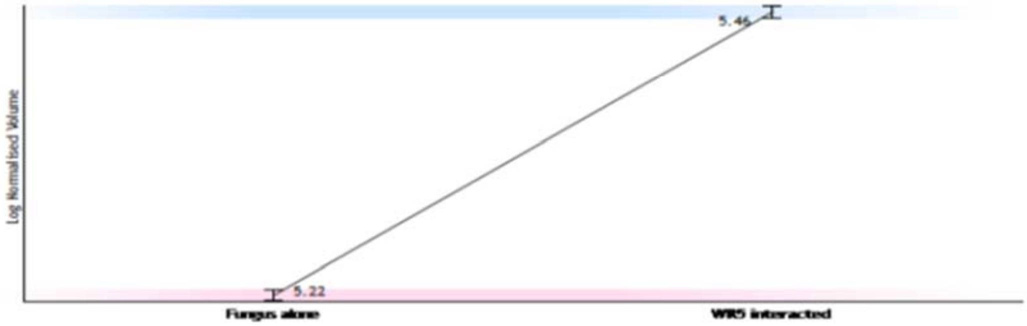

Identifier 320

Position (363, 651)
Notes

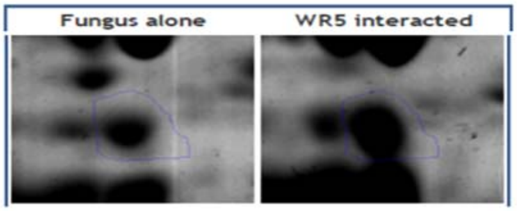

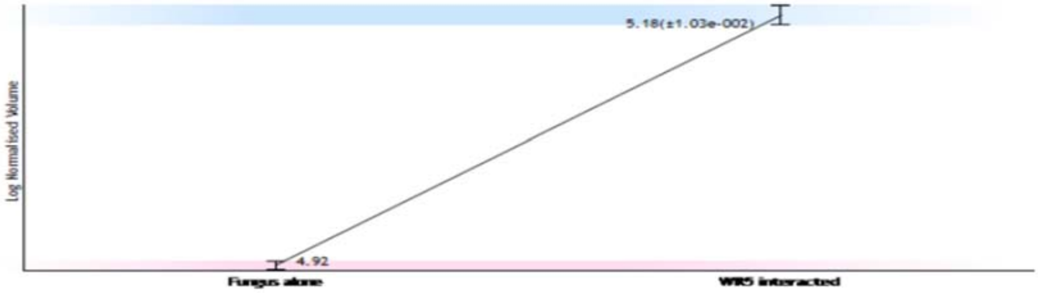

Identifier 342

Position (319, 724)

Notes

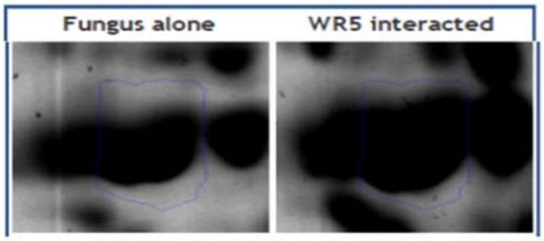

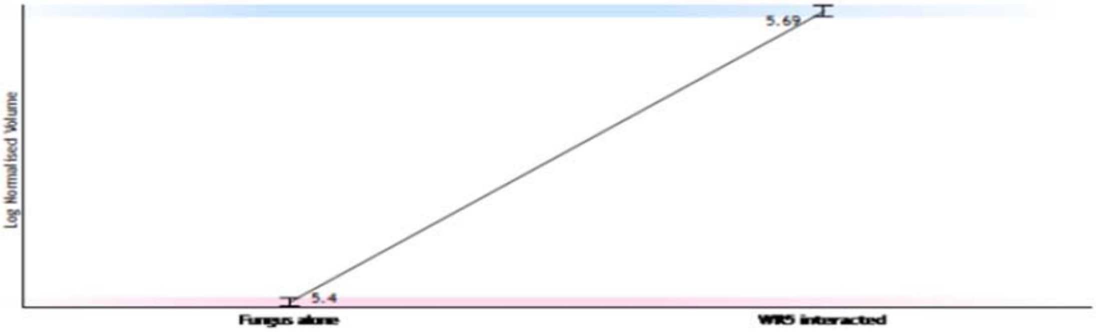

Identifier 287

Position (373, 583)

Notes

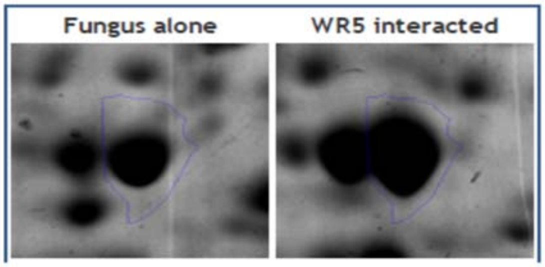

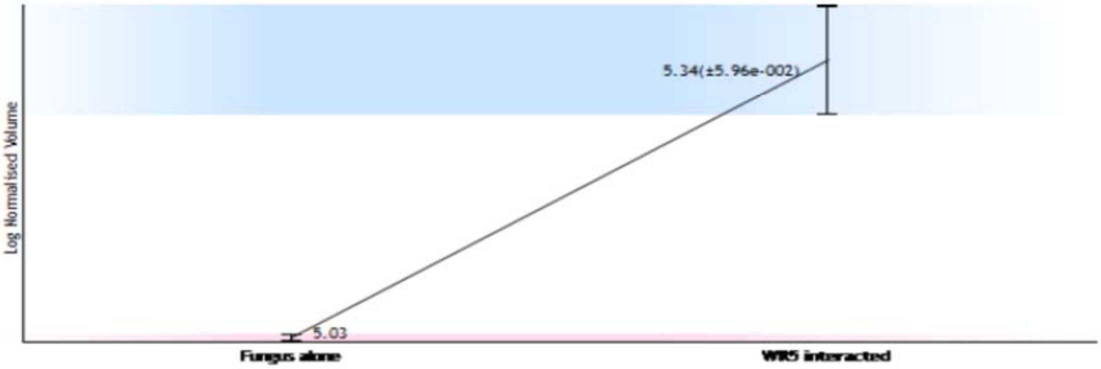

Identifier 428

Position (613, 1084)

Notes

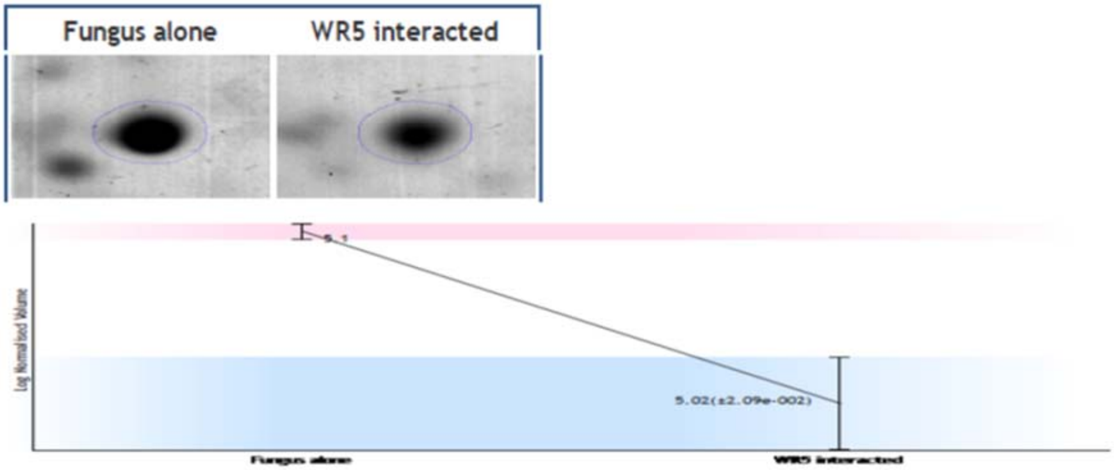

Identifier 330

Position (965, 701)

Notes

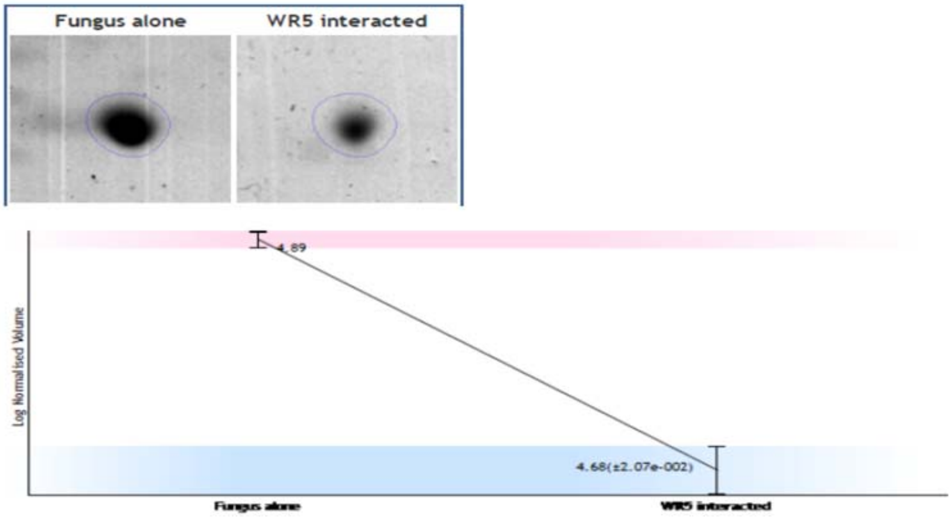

Identifier 305

Position (858, 623)

Notes

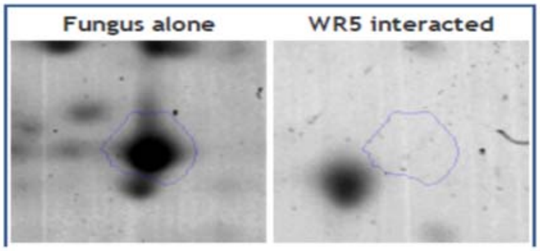

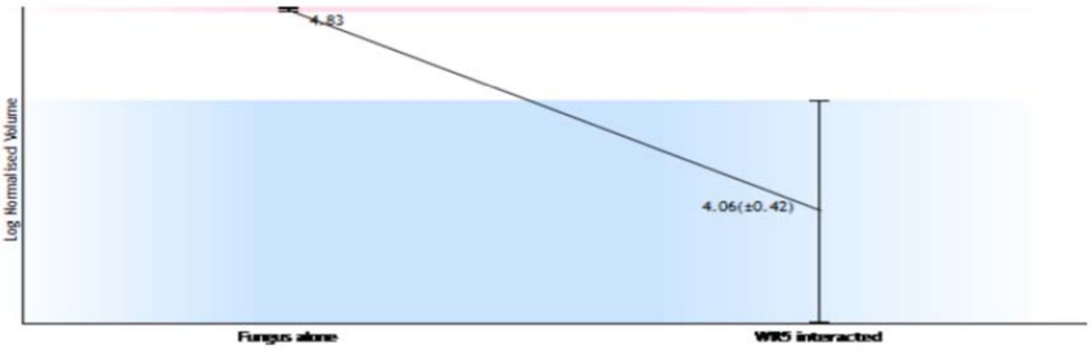

Identifier 296

Position (481, 606)

Notes

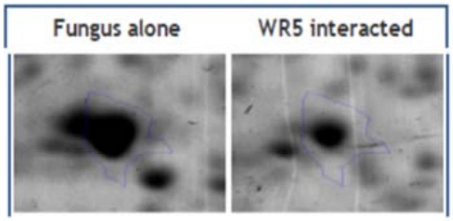

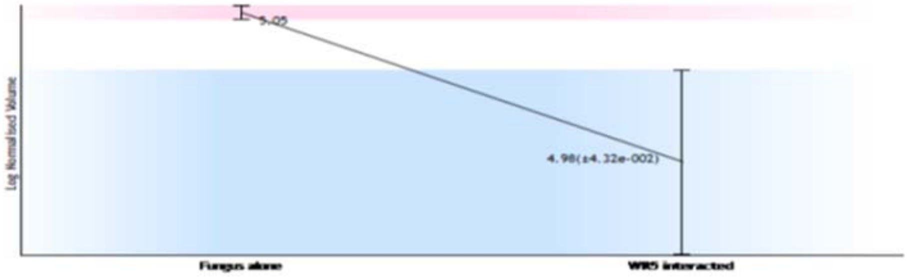

Identifier 207

Position (442, 427)
Notes

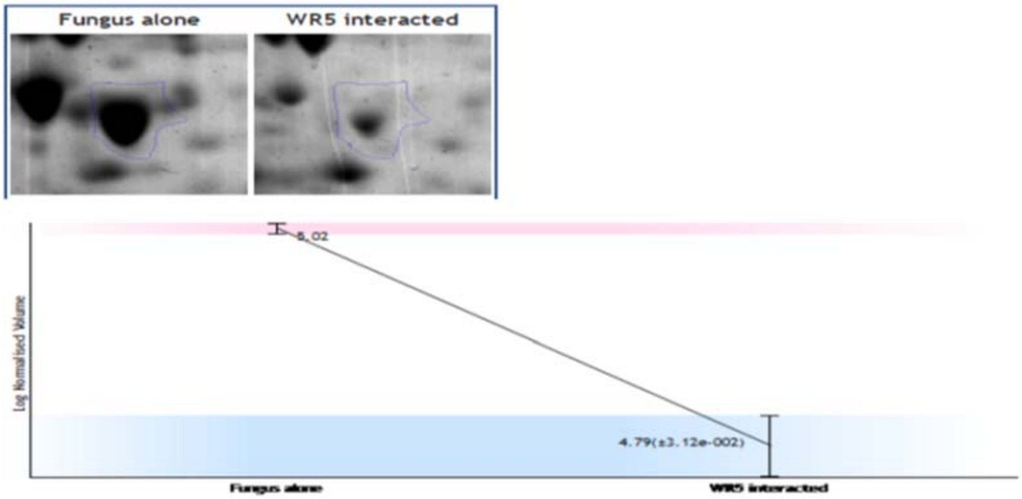

Identifier 198

Position (398, 406)
Notes

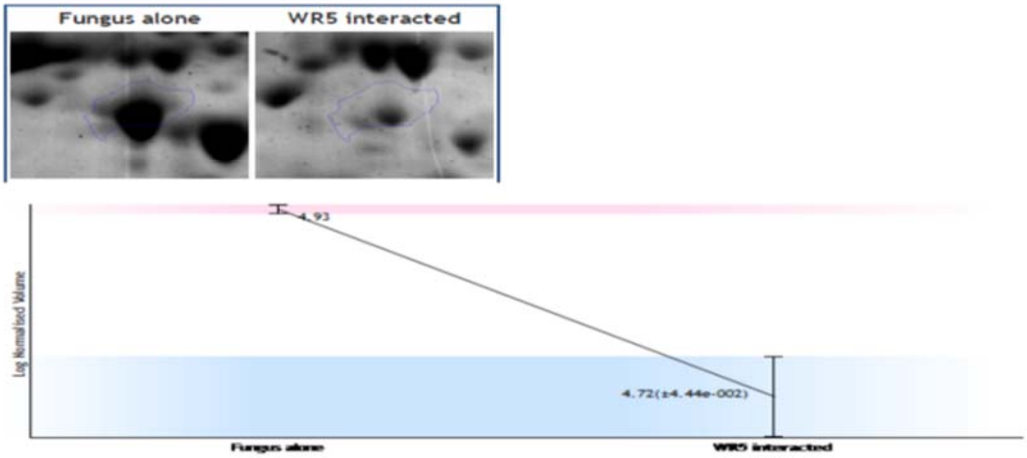

Figure S4. A list of protein spots identifier number, that exhibit a drastic change in expression fold level in M4-treated fungus analyzed pairwise between control and M4-treated *P. indica* using Progenesis SameSpots analysis software.

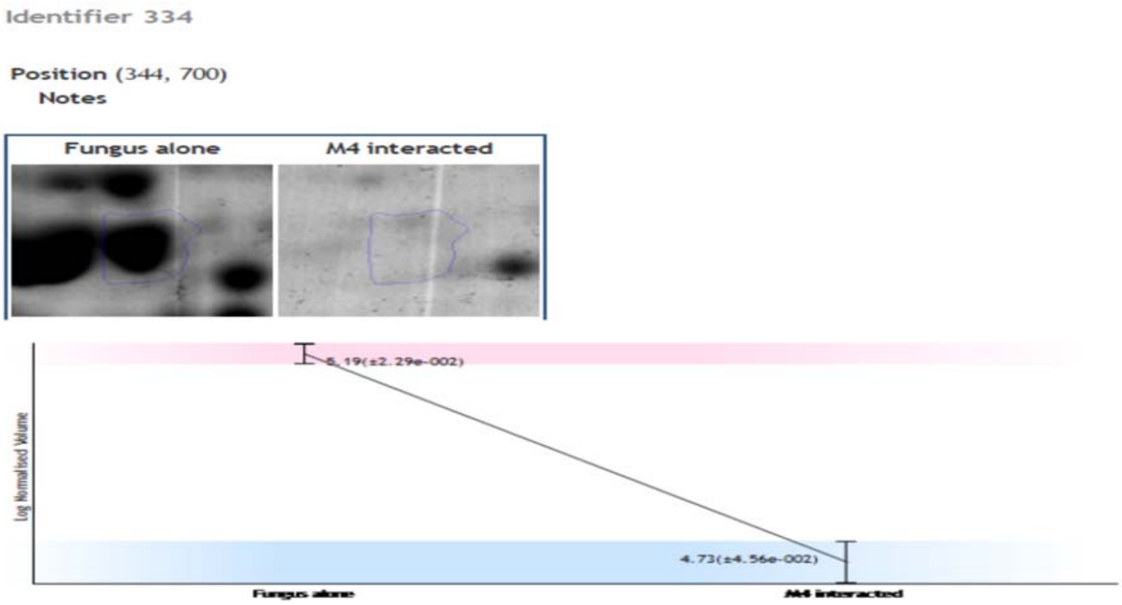

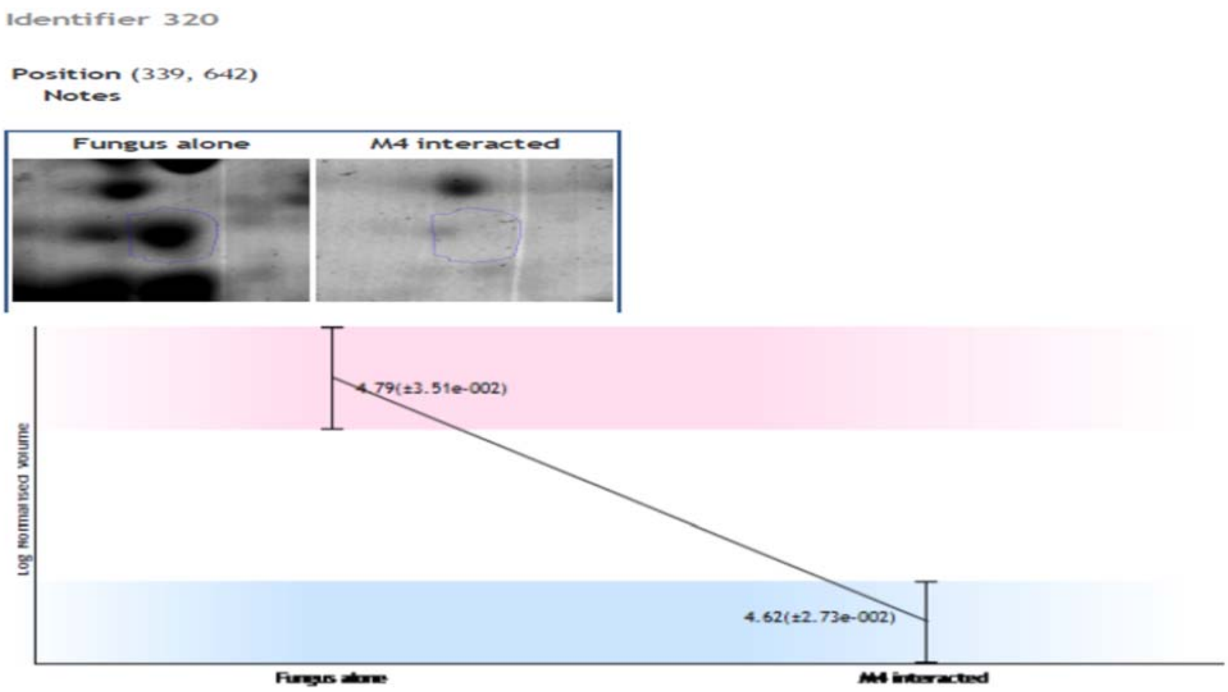

Identifier 341

Position (295, 716)

Notes

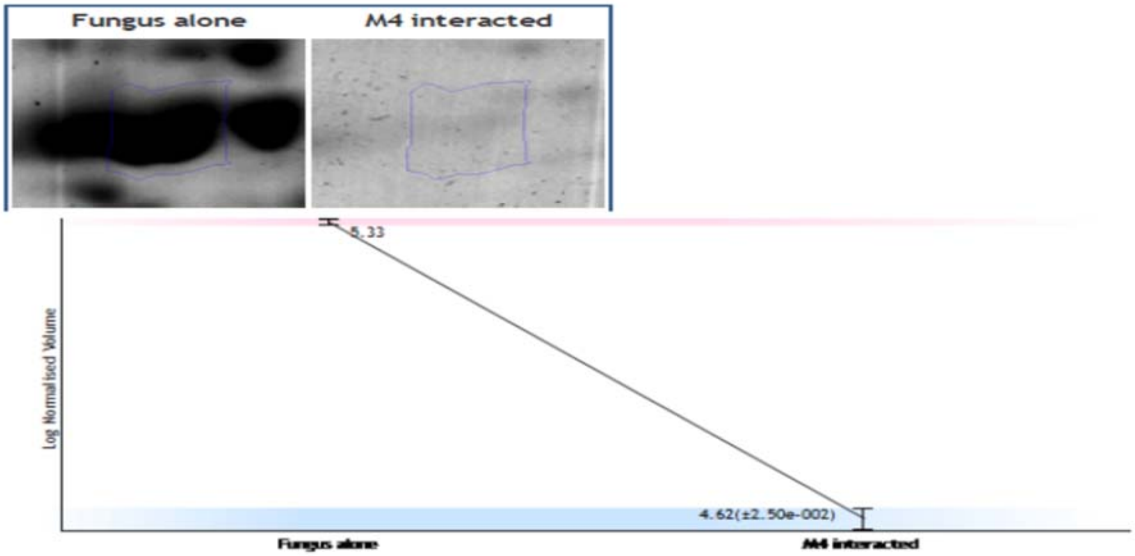

Identifier 292

Position (349, 574)

Notes

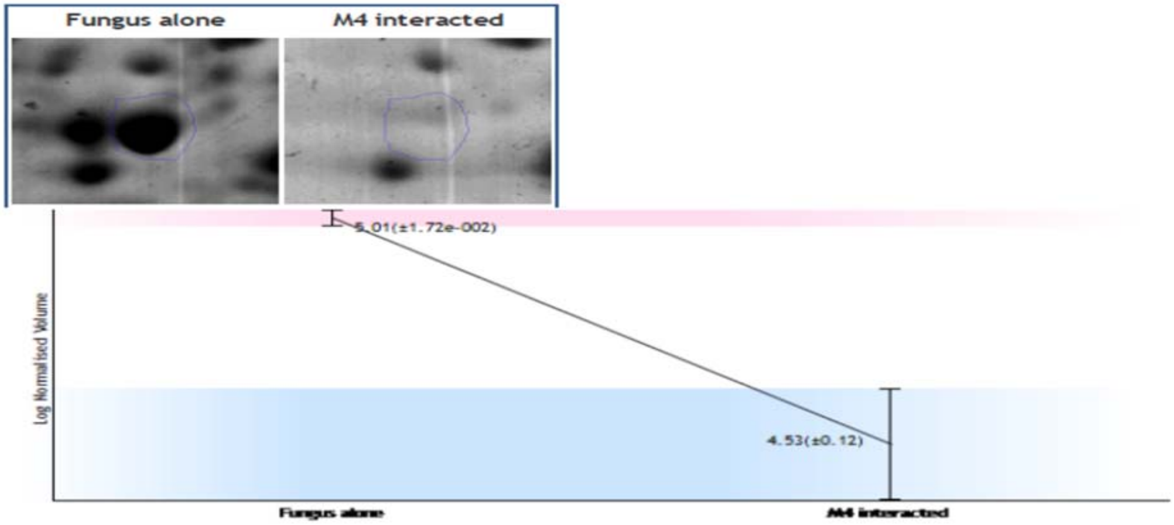

Identifier 452

Position (690, 1166)
Notes

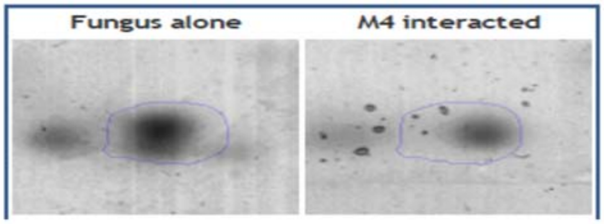

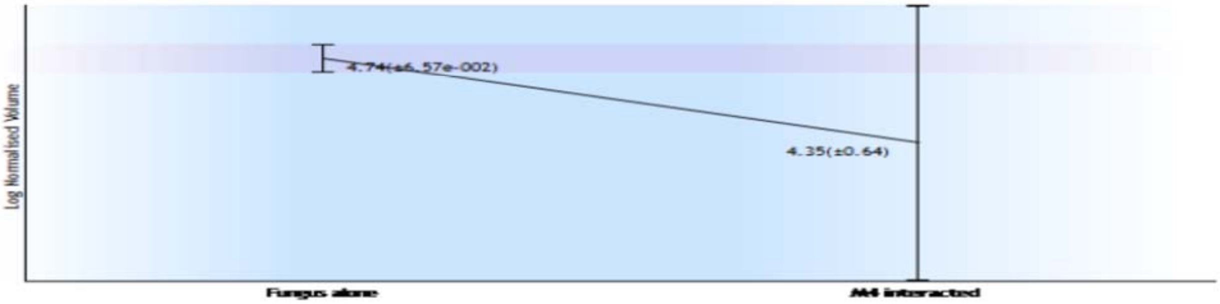

Identifier 422

Position (588, 1076)
Notes

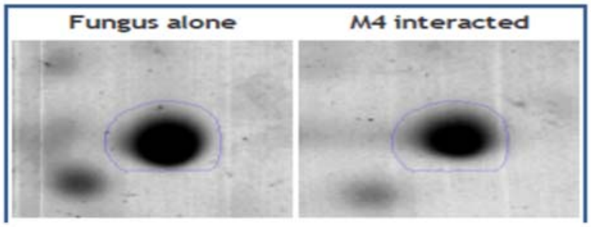

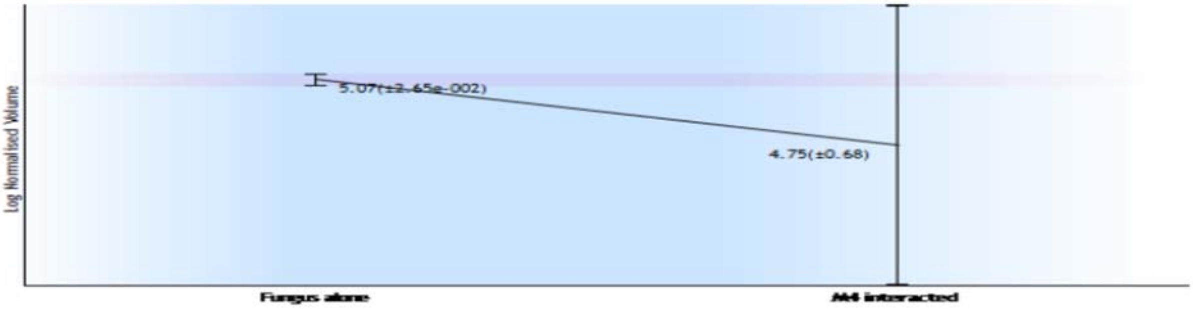

Identifier 309

Position (833, 614)
Notes

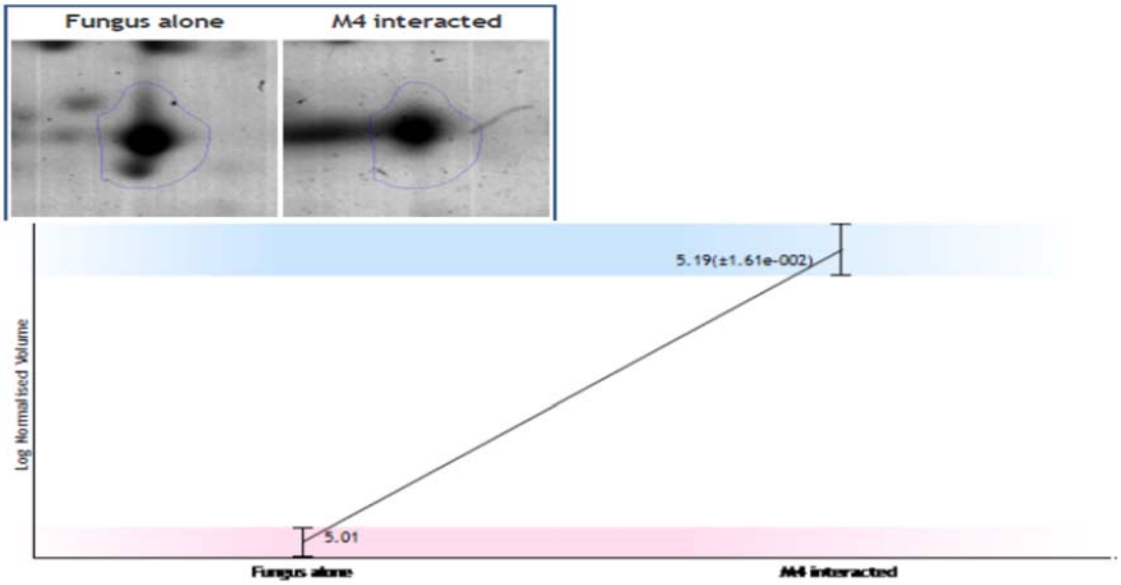

Identifier 301

Position (435, 602)
Notes

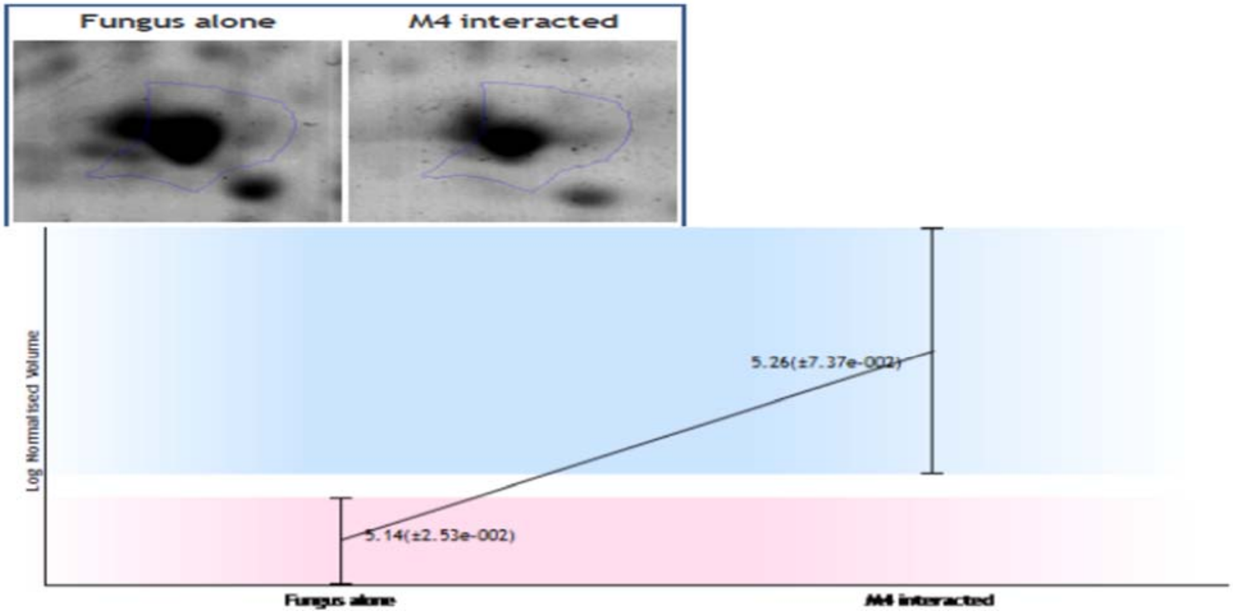

Identifier 210

Position (418, 420)
Notes

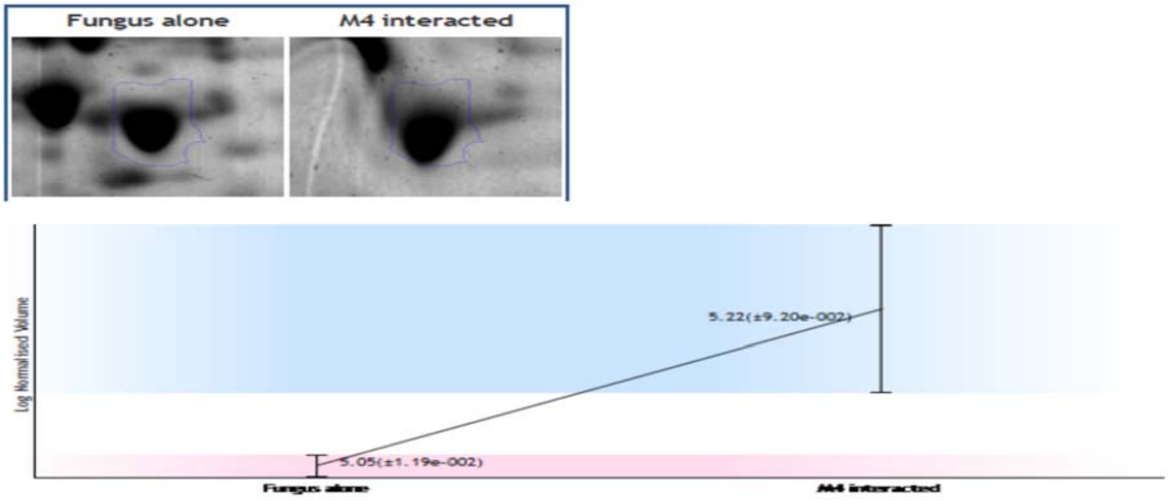

Identifier 198

Position (371, 402)
Notes

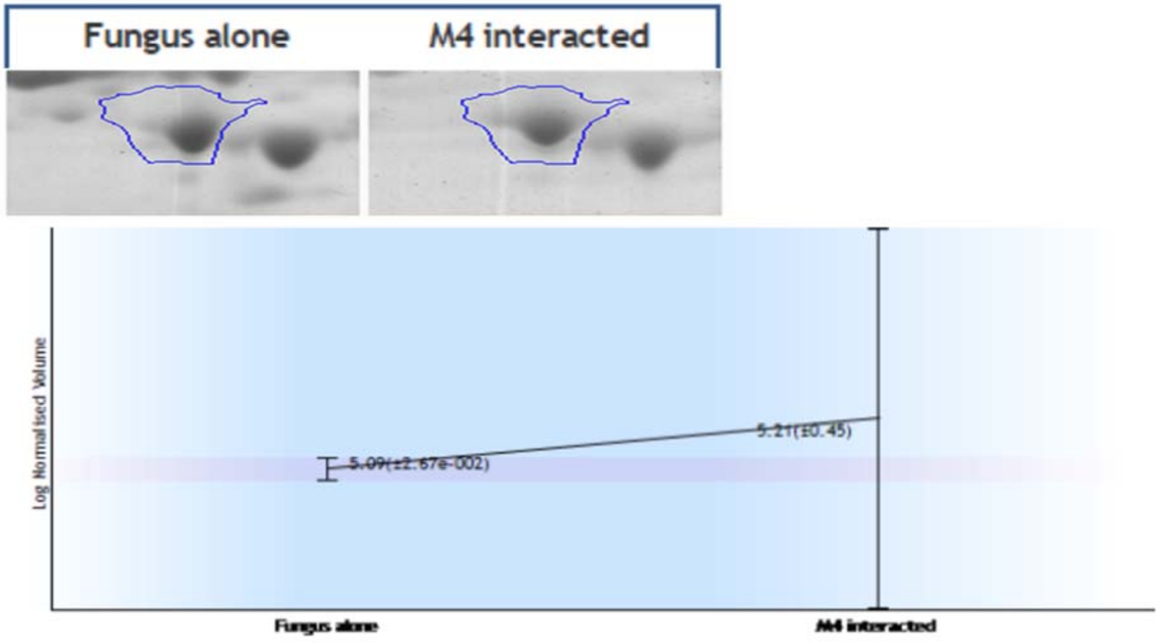

Figure S5. The matched peptide list of total sixteen protein spots identified through MALDI-TOF/MS of all three groups.

Spot number 3Wu

hypothetical protein PIIN_09126 [*Piriformospora indica* DSM 11827]

```
        10         20         30         40         50         60         70         80
MAAYLNRVVG SDFGGCSMNQ PYAMANRVNF DRGFVCGRRK DVEETKSYRQ LDPHPHLLIR YIRDTAGTLR INNPQTRRTI
        90        100        110
ATLARSTSKG ARSTHPPAFH PH
```

Spot number 4Wu/4M

hypothetical protein PIIN_04261 [*Piriformospora indica* DSM 11827]

```
        10         20         30         40         50         60         70         80
MARCVNSCYQ SSLLHITGFI SLVLIQGAPI DRAFKRRVKV DLHPLLQFKG GKAGIAIHTY RHTQSGFPPI NALLNLARFY
        90        100        110        120        130        140        150        160
AQTVAVLLAS SAAAVPMIDI HGLDYSDVGT ATFFLLTGGH TLPADQGTTE RDFDVSSSSA ILLRQDAPYW CPERFLSTAS
       170        180        190        200        210
FQWEQCTWFI WLHSLGDHRL KRLSAQGAPA STTVEHVLVR WKKL
```

Spot number 6Wu

related to YPT-1 GTP-binding protein ypt1 [*Piriformospora indica* DSM 11827]

```
        10         20         30         40         50         60         70         80
MAGDARPKVE ETFEPLHLLA TLTMHASEYD YLFKLLLIGD SGVGKSCLLL RFADDTYTES YISTIGVDFK IRTIELEGKT
        90        100        110        120        130        140        150        160
VKLQIWDTAG QERFRTITSS YYRGAHGIIV VYDVTDTDTF TNVKQWLQEI DRYASEGVNK LLVGNKSDLT SKKVVEYTVA
       170        180        190        200        210        220        230
KEFADQLGIP FLETSAKNAT NVEQAFLTMA KQIKDKMGAS GTGGPTSKTN TITPGTGITT NTSSGCC
```

Spot number 8Wu

hypothetical protein PIIN_10216 [*Piriformospora indica* DSM 11827]

10	20	30	40	50	60	70	80
MDIIVTTFSS	LSLGTILLLC	AIPWWPVIKI	AITLSFIVFP	VMLFLRHLRN	HPLSEGSTAY	LWSGLWILSY	TRPTTRIHKY
90	100	110	120	130	140	150	160
LQSNGVVRYR	LSRLPTEIWY	KILTFALDMP	EIMDSICTSE	IIWEEPDPDA	HQTTNRASIP	IHRRTESSAR	DTHATKARVE
170	180	190	200	210	220	230	
PSLLGVEQHS	AALSDRVVLR	PSGRLPHGQA	ARTDIGRHSY	ATKLWRVLNE	LDEGSRPSSL	AYPCHL	

Spot number 9Wu

predicted protein [*Laccaria bicolor* S238N-H82]

10	20	30	40	50	60	70	80
MLPHQLLLDQ	LPLVCEQDPC	ISVEYAWINQ	PSNNPLCQLS	TLKQVTFDLP	FKEWLIITPG	HAKLLQGIMS	DVGQATAEDG
90	100	110	120	130	140	150	160
HQWERLMEGE	KEVDIARQQS	LISLAKPVSM	ILLVNNQLTK	ITHLMMAFIF	NFSVSGTTLW	KETNQSKSCI	VKVKVAF

Spot number 11Wu

hypothetical protein PIIN_02973 [*Piriformospora indica* DSM 11827]

10	20	30	40	50	60	70	80
MVESAKRRWS	YRTFVDTAQV	LFGKAQVPQD	CMLAIQQTVE	LGRWKQNELD	KECLSYRTLA	RCALHLERLL	DQRLQLALLH
90	100	110	120	130	140	150	160
SSVLPAENPT	TKVEKSDFLQ	LYTHFNEDDM	LYQGTLEILM	QSPALTLALG	GHSWSGGESY	ISLDDVYPPL	APSSLLISSK
170	180	190	200	210	220	230	240
SQSQELESKT	LPSGESPVAL	PYLTALLFLH	AVVSGFSPNV	PEIMETTQRI	LVILRAPNQN	ITLLQRLPAT	ALVARLSEDK
250	260	270	280				
HAKELFDDLL	LRLEDPALVQ	TARLLSGLGE	PAESSHL				

Spot number 17Wu/17Fc

hypothetical protein PIIN_05768 [*Piriformospora indica* DSM 11827]

10	20	30	40	50	60	70	80
MSKSTLVPAR	NAIHGTGRAK	LVGFGTTASF	AELSYTYPLK	LLSPRQTTAT	NIERLAILYN	LSYGGGLIGG	DQLALTLELG
90	100	110	120	130	140	150	160
SDVKLLFLTQ	GSTKIFPSRA	RPRLATGRAE	VGDTRQRLSA	TIGSGACLVM	LPDPVTPFSH	SSYSQVQIFR	LAAPTKDTAG
170	180	190	200	210	220	230	240
GSIVLLDWFT	SGRMSRGEEW	QFDRYRSANE	IWIGNERVAS	DVLLLEQPPS	SSSVDETAKK	INRLRDSLAP	YACYATLLLF
250	260	270	280	290	300	310	320
GPATHALSKE	FAQRYDGLSQ	MQRKVPEDLI	WSYSPLEYKT	SINNGKRNSG	QKETGVGAIV	RVAAKDTESV	RQWLRESLRA
330	340						
GLEAQIGRNA	YSKAFV						

Spot number 18Wu/18Fc

u3 small nucleolar RNA-associated protein 11 [*Coprinopsis cinerea* okayama7#130]

10	20	30	40	50	60	70	80
MTSSLRNSLH	RRNHKERSQL	AHRTKLGFLE	KHKDYVKRAR	DYHSKQDRLN	RLKQKAAERN	KDEFYFSMKR	EKTRAGVHIK
90	100	110	120	130	140	150	160
DRGNAAIPTD	IVKVLKTQDE	NYVRTMRLSN	LKKIDRLKRQ	LTEMADLFKS	SLGGEDLEED	EYEVLQEAGI	LPPSGKKRGR
170	180	190	200	210	220	230	240
SKSKHLVFAE	SLEEAQTLGQ	KAKTAAEPDH	SSPPQEPEPT	PEDLGWKTRD	NKKKRRKTRQ	TIEELEDVDG	EWEDEDDLTT
250	260	270	280	290	300	310	320
HDSGSQSSTE	KRTRLLKEIS	ARLVRDRQLR	YTQREFEMQR	LLMGKGAAKK	IAGVEKFGED	DDSEDDEDAL	DARGGRPLKK
330	340	350					
SKVVDEATYK	PRVYKWKLER	KR					

Spot number 19Wu/19Fc

related to RTM1 protein [*Piriformospora indica* DSM 11827]

10	20	30	40	50	60	70	80
MERSYFPYKA	SKPAAVLFAV	LYSVSVAAHV	FQMIKLRAYF	MSVLIVGLLV	EVLGYVTRKL	AIDDDPTLWS	FSTSQVCILV
90	100	110	120	130	140	150	160
APAFLAASAY	MIVGRMMAYV	GPGASVISHR	LITKVFVIVD	ILCLITQAAG	IAMFVTNVDK	ADRTVVLRGR	NILMTGLALQ
170	180	190	200	210	220	230	240
IISYLIFVVI	TIIFDIRAQR	MKGTQLKKLR	PLFWASYSVA	FLIIGRSIYR	AIEFGTVDFK	RRTQGYLYTH	EWPFYVLDAV
250	260	270	280	290	300		
PILAAAVILN	VIHPSSYLPS	KKGLRMDGTI	EVPARHWWSK	SKSTKEHELE	NATP		

Spot number 21Wu/21Fc

probable ENO1-enolase I (2-phosphoglycerate dehydratase) [*Piriformospora indica* DSM 11827]

10	20	30	40	50	60	70	80
MSITKVHARQ	IFDSRGNPTV	EVDLYTAKGR	FRAAVPSGAS	TGVHEAVELR	DGDKKSYVGK	GVLNAVKNVN	ETIAPALIDS
90	100	110	120	130	140	150	160
GLSVTQQKDI	DALLIKLDGT	PNKGKLGANA	ILGVSIAVAE	AGAAESGLPL	YAYLAKLAGH	PEGGKMTMPC	PAFNVINGGS
170	180	190	200	210	220	230	240
HAGNGLAFQE	FMLLPTGATS	FTEAMKIGTE	TYHTLKKVIQ	AKYGLDATNV	GDEGGFAPNV	AGAEESLELL	SEAIKKAGYE
250	260	270	280	290	300	310	320
GKIKIGLDVA	SSEFYKDGKY	DLDFKNANSD	PTKWITGKEL	GDFYNKMIEK	YPIVSIEDPF	DQDDWEAWSS	FTNGTKVQVV
330	340	350	360	370	380	390	400
GDDLTVTNPE	RIQTAIGKKA	CNGLLLKINQ	IGTISESIQA	AQLAQSDGWG	VMVSHRSGET	ENTVIADLVV	ALGGCQIKTG
410	420						
APARSERVAK							

Spot number 5Wd/5M

G protein alpha-subunit [*Laccaria bicolor* S238N-H82]

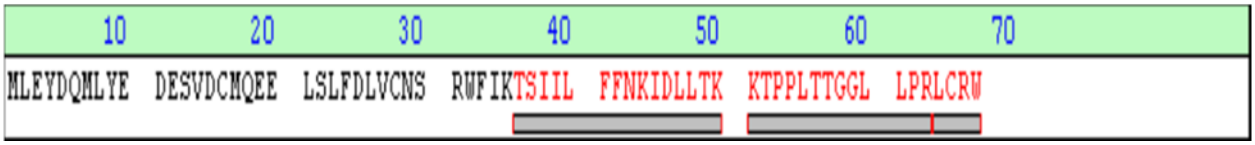

Spot number 12Wd/12M

predicted protein [*Laccaria bicolor* S238N-H82]

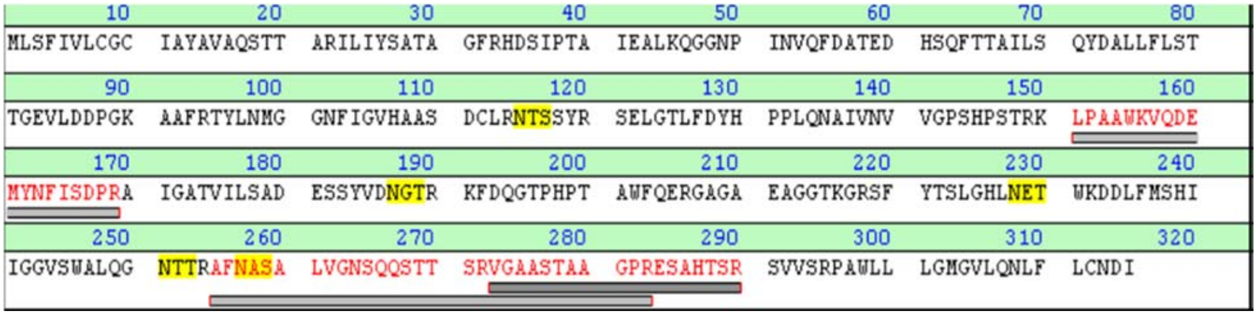

Spot number 13Wd/13Mu

predicted protein [*Laccaria bicolor* S238N-H82]

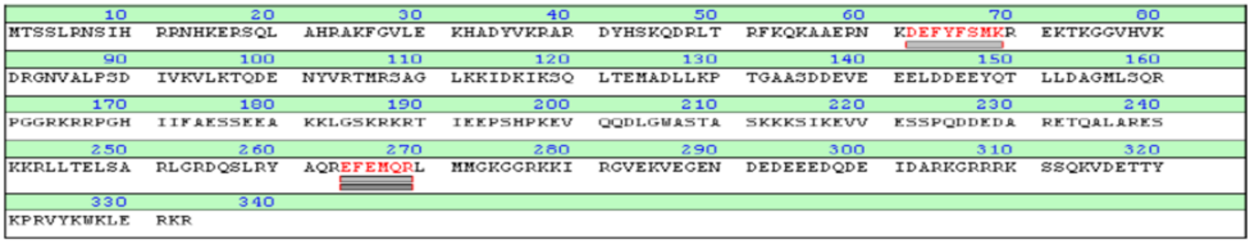

Spot number 20Wd/20Mu

hypothetical protein PIIN_01661 [*Piriformospora indica* DSM 11827]

10	20	30	40	50	60	70	80
MNTPQRRKRV	GSPAEGDMSG	LSAGIGVELS	MEDKERLIEN	LDIEVTNHRR	RFEAFLEQLL	EGFSLRHGME	ISRIPKAIRN
90	100	110	120	130	140	150	160
MKMKDFDAFG	GNVQRCVQAM	ARQRVAEADG	DANAKKRKWQ	AAIQEEEIER	ASKTPRVAPP	SPVKTSKPSS	QVPNSSRAKG
170	180	190	200	210	220	230	240
LFPKTPTRNM	NNVNPTKTAL	HGSARKVMPS	PSKRAVSPTK	PQNMPTSSTS	SFKPASTTFN	PVLPPKTPTY	PRRVARADEA
250	260	270	280	290	300	310	320
VMSLNGTPLK	FPSAFMFPRG	VAPIVDEGEE	SEHDSHAKST	GVGPAFLVRR	DPSTVSMGSS	SASTAVSSTG	TLITISTSKG
330	340	350	360	370			
QTVQFDPLMA	SPGSLDRIPD	LTESAKKQVR	EDTARIVQAL	HKWRL			

Spot number 22Wd/22Mu

hypothetical protein PIIN_01521 [Piriformospora indica DSM 11827]

10	20	30	40	50	60	70	80
MASVIPSVTP	NARSTLQREY	FTSELFVNPA	RSDIEDLLAS	FEEAVEQPLK	PFDIFKTVWS	QEGWKFTCLY	IWEDTARDQY
90	100	110	120	130	140	150	160
LQTMLRLFLE	RVKDSESFAR	QLGAVFGLYT	FFQQQATIKS	LYSVSSIEIT	IDKLEYLCSL	CKESPVAHQP	YYVFILRYFL
170	180	190	200	210	220	230	240
ERDIFHILPH	SSLQPYNPTR	LPASFVVSRE	SSSGADASSH	GGNSSKTTRG	GRTKAERDRY	GREQWAQLGS	LVRLQRQRQK
250	260	270	280	290	300	310	320
GEVDDVEIAV	GAQPHSDANK	NTSGSTPFPD	KDSSAVVYRE	AKSRVLEVLP	KEVEREAEQR	ALQRLKTLAS	SVESDVHSGL
330	340	350	360	370	380	390	400
KKQRLMETDD	SMMEMESVTA	AQKDNQRQRE	EGDDEGGMVS	QAHKVSHASD	GVARLENMIK	NGHGVLRNQW	ANE

Spot number 23Wd/23M

hypothetical protein PIIN_03309 [*Piriformospora indica* DSM 11827]

10	20	30	40	50	60	70	80
MFYGHPEHQP	NAANLLWELI	EQDERALQEV	KEEEERLENE	REGILAHLGV	LANDTQVLGQ	LAEVALPNIK	NIIDALKPEI
90	100	110	120	130	140	150	160
SALETKREDG	HFSNYGGWLE	KYKNYIKVTV	GFMEEIQHHK	TQQLVREYVE	PESMARLKLD	HIDGLLKETA	DRIQATSIRL
170	180	190	200	210	220	230	240
QTKRLKLSKT	PFSRLTDENW	RLIFGYASIS	FHPHHAGEQV	CDRSISTSIH	ALTRVCHRWY	KVIYQSSKLW	RRVYLNYEGL
250	260	270	280	290	300	310	320
KNDSSRKLLN	LILSKAAEYV	GHILDALPTP	YELAIRSLLM	TNTRLLYHPR	SLDTLEVVEA	TNVELVLETG	RNLPNLKLLK
330	340	350	360	370	380	390	400
LQANTSITPC	TEFYDTYSKT	STIDLAGGSF	HLPRRVPRLQ	PWDAHSIRGT	LNSILSVFSP	RVVFTQLSEI	TLRFPPEPAN
410	420	430	440	450	460	470	480
MPDLATWCSF	LSSSKASTVT	SLTLYPMENA	KILSHYVTAL	HNLARLRVHG	SSVDPLLKEL	TWQVEDERSK	VLTFLSVLEI
490	500	510	520	530			
HDYDGRGSPV	VEFYOSKLAS	SYRTLRTVRF	ANCPNIPOEI	ROIFIDN			
